# Characterization of Flavin-Based Fluorescent Proteins: An Emerging Class of Fluorescent Reporters

**DOI:** 10.1371/journal.pone.0064753

**Published:** 2013-05-31

**Authors:** Arnab Mukherjee, Joshua Walker, Kevin B. Weyant, Charles M. Schroeder

**Affiliations:** 1 Department of Chemical and Biomolecular Engineering, University of Illinois at Urbana-Champaign, Urbana, Illinois, United States of America; 2 Center for Biophysics and Computational Biology, University of Illinois at Urbana-Champaign, Urbana, Illinois, United States of America; 3 Department of Materials Science and Engineering, University of Illinois at Urbana-Champaign, Urbana, Illinois, United States of America; 4 Department of Chemistry, University of Illinois at Urbana-Champaign, Urbana, Illinois, United States of America; University of Illinois, Urbana-Champaign, United States of America

## Abstract

Fluorescent reporter proteins based on flavin-binding photosensors were recently developed as a new class of genetically encoded probes characterized by small size and oxygen-independent maturation of fluorescence. Flavin-based fluorescent proteins (FbFPs) address two major limitations associated with existing fluorescent reporters derived from the green fluorescent protein (GFP)–namely, the overall large size and oxygen-dependent maturation of fluorescence of GFP. However, FbFPs are at a nascent stage of development and have been utilized in only a handful of biological studies. Importantly, a full understanding of the performance and properties of FbFPs as a practical set of biological probes is lacking. In this work, we extensively characterize three FbFPs isolated from *Pseudomonas putida*, *Bacillus subtilis*, and *Arabidopsis thaliana,* using *in vitro* studies to assess probe brightness, oligomeric state, maturation time, fraction of fluorescent holoprotein, pH tolerance, redox sensitivity, and thermal stability. Furthermore, we validate FbFPs as stable molecular tags using *in vivo* studies by constructing a series of FbFP-based transcriptional constructs to probe promoter activity in *Escherichia coli*. Overall, FbFPs show key advantages as broad-spectrum biological reporters including robust pH tolerance (4–11), thermal stability (up to 60°C), and rapid maturation of fluorescence (<3 min.). In addition, the FbFP derived from *Arabidopsis thaliana* (iLOV) emerged as a stable and nonperturbative reporter of promoter activity in *Escherichia coli*. Our results demonstrate that FbFP-based reporters have the potential to address key limitations associated with the use of GFP, such as pH-sensitive fluorescence and slow kinetics of fluorescence maturation (10–40 minutes for half maximal fluorescence recovery). From this view, FbFPs represent a useful new addition to the fluorescent reporter protein palette, and our results constitute an important framework to enable researchers to implement and further engineer improved FbFP-based reporters with enhanced brightness and tighter flavin binding, which will maximize their potential benefits.

## Background

Beginning with the discovery of the green fluorescent protein (GFP) and its subsequent heterologous expression in diverse organisms, fluorescent reporter proteins have been established as integral components of the molecular biology toolkit [1]–[4]. GFP-based fluorescent proteins have been identified, isolated, and characterized from several distinct sources, including *Aequorea victoria*, *Renilla reniformis*, *Anthozoa* sp., and *Discosoma* sp., [2], [5]–[9]. However, all GFP-based reporters show oxygen-dependent fluorescence [1], [10]–[12]. Consequently, GFP-based fluorescent proteins are dimly fluorescent to nonfluorescent in low-oxygen environments [13]–[16], which has precluded their application for investigating bioprocesses that are regulated by hypoxia and anoxia such as microbial fermentation, bioremediation, anaerobic sewage treatment, tumor metastasis, development of chronic inflammation [17], cerebral hypoxia-ischemia, microbial pathogenesis, and biofilm formation.

**Figure 1:**
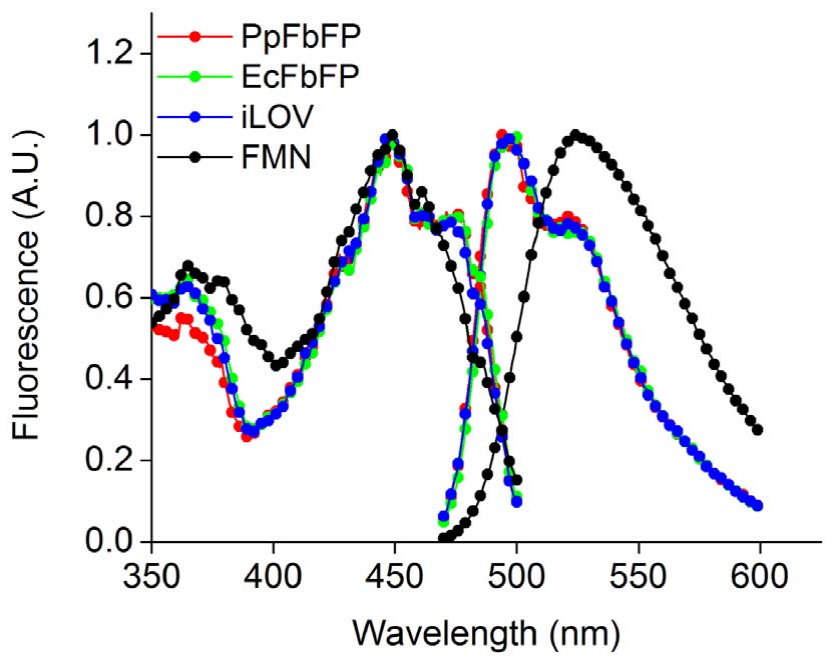
Excitation and emission spectra of FbFPs and FMN. Purified FbFPs and FMN in anion exchange elution buffer (20 mM Tris hydrochloride, 1 M sodium chloride, pH 8.0) were excited with 450 nm light, and the emission spectra were recorded by scanning emission between 480 and 600 nm. Excitation spectra were recorded by monitoring emission at 520 nm and scanning the excitation wavelength from 350 to 500 nm.

Recently, a new class of oxygen-independent fluorescent reporters based on bacterial and plant photosensory flavoproteins has been developed [18], [19]. These photosensory proteins share a highly conserved light, oxygen, or voltage (LOV) sensing domain that binds flavin mononucleotide (FMN), which is a UV-A/blue light sensing fluorescent cofactor [20], [21]. Excitation of these reporter proteins with blue light at 450 nm results in cyan fluorescence emission with a peak at 495 nm (**Figure 1**). Specifically, three members of the LOV-family of proteins have been identified as FMN-based fluorescent proteins (FbFPs): (1) PpFbFP, which is based on an uncharacterized sensory box protein from *P. putida* [19], (2) EcFbFP, which is an *Escherichia coli* codon-optimized gene [19] derived from the N-terminal LOV domain of the *Bacillus subtilis* YTVA protein implicated in regulating stress response [22], [23], and (3) iLOV [18], which comprises the LOV2 domain of the *Arabidopsis thaliana* blue light photoreceptor phototropin (Phot2) that mediates diverse light driven adaptive responses including phototropism, stomatal opening, and chloroplast translocation [24], [25].

In their native context, LOV-domain proteins function as blue light photoreceptors that exhibit an elaborate photocycle upon absorption of blue light, involving: (1) conversion of the buried FMN to an excited singlet state, followed by immediate conversion to an excited triplet state by intersystem crossing with a red-shifted absorption peak at 660 nm, (2) decay of the excited triplet state to a singlet state that forms a covalent adduct between FMN and a neighboring cysteine residue in the protein, which results in a blue-shifted absorption peak at 390 nm, (3) eventual return to the dark ground state by thermal relaxation [26]–[29]. Repeated cycling of the photostimulated protein between ground and excited states results in bleaching of fluorescence [18], [30]. In order to engineer fluorescent variants of the LOV proteins, the photochemical cycle was abolished through mutation of the active site cysteine residue to alanine. The Cys to Ala mutation prevents the formation of the covalent adduct between the protein and excited state FMN, thereby providing a molecular framework to engineer FbFPs [18], [19].

FbFPs from *P. putida* (PpFbFP) and *B. subtilis* (codon optimized for expression in *E. coli* and subsequently designated EcFbFP) have been shown to express and fluoresce in anaerobically cultivated *E. coli* cells under conditions in which the GFP-based yellow fluorescent protein (YFP) was nonfluorescent [19]. Furthermore, EcFbFP was shown to outperform YFP as a fluorescent reporter for real time monitoring of gene expression in *E. coli* cells cultivated using rich media. Specifically, fluctuations in oxygen levels occurred as the *E. coli* cells transitioned from actively respiring exponential phase of growth to the stationary phase, thereby resulting in inaccurate assessment of promoter activity by the oxygen-dependent fluorescence of YFP. In contrast, excellent agreement was obtained between fluorescence levels of EcFbFP (derived from *B. subtilis* and codon-optimized for expression in *E.coli*) and mRNA measurements by reverse transcription PCR [30]. Heterologous expression and fluorescence of FbFPs has been demonstrated in facultative and obligate anaerobes including *Rhodobacter capsulatus* [19], *Porphyromonas gingivalis* [31], *Bacteroides fragilis* [32], *Roseobacter, Phaeobacter* spp. [33], *Candida albicans*, and *Saccharomyces cerevisiae* [34], as well as in animal cells [35]. In a recent study, a translational fusion between oxygen-independent EcFbFP and oxygen-sensitive YFP was utilized as a real-time intracellular reporter of oxygen concentration in the cytoplasm of *E. coli* cells [36].

In addition to oxygen-independent fluorescence, FbFPs are also characterized by small size (∼100–140 amino acids), which is a significant advantage over bulkier GFP-based probes (∼240 amino acids) for generating minimally perturbative translational fusions. Chapman *et al.* capitalized on this key advantage by using the *A. thaliana* FbFP (iLOV) to track the development of local and systemic infection by the tobacco mosaic virus in tobacco leaves [18]. In this study, the authors observed that fusions to bulkier GFP molecules hindered effective viral invasion leading to the development of localized viral lesions. Finally, spectrally improved variants of existing FbFPs have been engineered using directed evolution. Christie *et al.* employed DNA shuffling to enhance brightness and photostability of iLOV [18], [37]. Recently, we discovered brighter mutants of PpFbFP by saturation mutagenesis of chromophore-proximal amino acids [38]. In this work, substitution of a chromophore proximal aromatic amino acid at position 37 (phenylalanine) by serine or threonine (Phe37Ser and Phe37Thr mutants) enhanced brightness of fluorescence emission by nearly 74%. Specifically, the mutations improved quantum yield by relieving fluorescence quenching interactions between FMN and Phe37 and strengthening association between the protein and the FMN chromophore.

In order to expand the scope and enhance the utility of FbFPs as practical fluorescent reporters, a comprehensive evaluation of the performance properties of FbFPs is critically required. In this work, we systematically evaluated the existing set of FbFPs with respect to quantum yield, oligomeric state, holoprotein fraction, and maturation time. Moreover, we investigated the effects of pH, temperature, and reducing conditions on FbFP fluorescence. Finally, we assessed the utility of FbFPs as reporters of promoter activity in wild type *E. coli* MG1655 cells expressing transcriptional fusions between individual FbFPs and inducible or constitutive bacteriophage promoters. Aside from the oxygen-independent function of these reporter proteins, we identified several advantages to using FbFPs as reporters, including thermal tolerance (up to 60°C), fluorescence emission over a broad pH range (4–11), and rapid maturation of fluorescence (≈10–30 minutes in GFP *vs*. 1 minute in FbFPs). Based on our results, iLOV emerged as a suitable transcriptional reporter for *E. coli* and showed good agreement with transcriptional profiles determined using a YFP reporter as a benchmark. Overall, we anticipate that our results will enable broader application of FbFPs, as well as providing a basic biochemical framework to further engineer and optimize FbFPs as robust biological reporters.

## Results and Discussion

### Purification of FMN-based Fluorescent Proteins (FbFPs)

**Figure 2:**
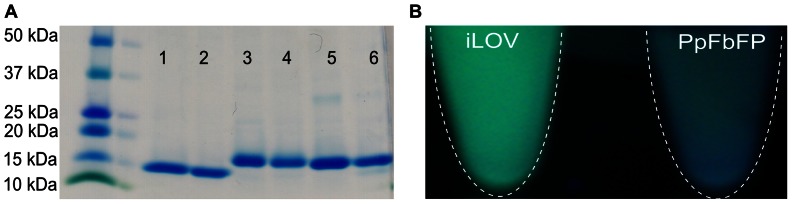
SDS PAGE analysis of purified FbFPs. FbFPs were purified using nickel affinity and anion exchange chromatography. A) Purified FbFPs were loaded on a denaturing polyacrylamide gel and allowed to migrate at 160 V for 40 min. Lane 1: iLOV after nickel affinity chromatography, lane 2: iLOV after anion exchange chromatography, lane 3: PpFbFP after nickel affinity chromatography, lane 4: PpFbFP after anion exchange chromatography, lane 5: EcFbFP after nickel affinity chromatography, lane 6: EcFbFP after anion exchange chromatography. B) Purified PpFbFP and iLOV emit cyan-green fluorescence upon excitation using a UV transilluminator.

In this work, we characterized the performance of FbFPs under a wide range of experimental conditions. In particular, we studied three distinct FbFPs originally derived from three different organisms: (1) PpFbFP isolated from *P. putida*, (2) EcFbFP isolated from *B. subtilis* and codon optimized for expression in *E.coli*, and (3) iLOV isolated from *A. thaliana*. FbFPs were purified using two-stage chromatography consisting of immobilized nickel affinity and anion exchange chromatography. Upon excitation with blue light at 450 nm, all three FbFPs displayed their characteristic emission spectra with a peak at 495 nm and a shoulder at 525 nm (**Figure 1**). In all cases, FbFPs were purified to greater than 95% homogeneity (**Figure 2**). Freshly purified FbFPs (average concentration 1–2 mg/mL) were used for all characterization experiments, and purified preparations were stable for at least a week at 4°C, as verified by fluorescence measurements on freshly prepared and stored samples.

### Quantum Yield of FbFPs

In a first set of experiments, we determined quantum yields (*QY*s) for all three FbFPs using FMN as a standard with known quantum yield (*QY_FMN_* = 0.27) [39]. Specifically, quantum yield was calculated by integrating the fluorescence emission spectrum between 480 nm and 600 nm and by normalizing the integrated emission by absorbance at the excitation wavelength (450 nm). In this way, quantum yield quantifies the efficiency with which flavin-bound FbFP molecules (holoprotein) convert excitation light into fluorescence emission. Quantum yields are provided in **Table 1** and are in agreement with previously determined values of quantum yields for these fluorescent proteins [18], [19]. We further determined the brightness of these fluorescent proteins, where brightness is defined as the product of quantum yield and molar extinction coefficient of flavin (ε = 12,500 M^−1^ cm^−1^) [19]. Based on these values, we identify EcFbFP and iLOV (brightness = 4250) as the brightest members of the FbFP family.

**Table 1:** Quantum yield and brightness of FbFPs.

Protein	Quantum yield	Molar extinction coefficient (M^−1^ cm^−1^)	Brightness
PpFbFP	0.17	12500	2125
EcFbFP	0.34	12500	4250
iLOV	0.34	12500	4250

Brightness is quantified as the product of quantum yield and molar extinction coefficient of the fluorophore (flavin mononucleotide). Brightness values are reported for monomeric units of PpFbFP and EcFbFP, which exist as functional dimers.

### Fraction of Fluorescent Holoprotein

FbFP fluoresce is facilitated by an FMN fluorophore that is noncovalently associated with the protein via an extensive network of hydrogen bonds, coulombic forces, and van der Waals interactions [37]. As a result, maturation of fluorescence in FbFPs relies on the availability of a sufficient pool of intracellular FMN. Moreover, the brightness of FbFPs is also a function of the strength of association between the protein and FMN. Therefore, in addition to the quantum yield and molar extinction coefficient, an accurate assessment of FbFP brightness needs to account for the fraction of FbFP in solution that is fluorescent, *i.e.*, the FMN-bound holoprotein fraction. We verified that apo-FbFPs do not substantially contribute to absorbance at 450 nm (**Text S1**). Furthermore, we found that the concentration of free FMN in a solution of purified FbFP is negligible (**Text S1**). Therefore, absorbance at 450 nm can be specifically attributed to the holoprotein species in solution. Based on these assumptions, we determined the fraction of fluorescent holoprotein in solution, defined as the ratio of holoprotein concentration to the total protein concentration (holo+apoprotein, estimated by the Bradford assay) (**Materials and Methods**). In recent work, we used an analogous approach to estimate holoprotein fractions in wild type and brightness enhanced mutants of PpFbFP [38].

Holoprotein fractions for the three FbFPs are presented in **Table 2**. We determined that EcFbFP (*f_holo_* = 0.67) surpasses the fraction of holoprotein in solution compared to PpFbFP (*f_holo_* = 0.59) and iLOV (*f_holo_* = 0.39). In order to explain the improved association with FMN in EcFbFP over iLOV, we examined the FMN-binding cavities in the two proteins. The structure of iLOV was recently solved (PDB ID: 4EES). In the absence of a crystal structure for EcFbFP, we used the crystal structure of a closely related protein, YTVA from *Bacillus subtilis* (PDB ID: 2PR5). The YTVA crystal structure was solved in the dark state conformation of the protein in which the FMN ring is noncovalently buried in the binding pocket. Therefore, the dark structure can be considered to closely resemble the EcFbFP structure in which covalent association between FMN and the protein is abolished through a C62A mutation. We defined the FMN binding cavity to include amino acids that are directly involved in hydrogen bonding or hydrophobic interactions with the isoalloxazine ring of FMN, and these amino acids were identified using Ligand Explorer version 4.0 (**Text S2**). Despite a high degree of similarity between amino acids comprising the binding sites in the two proteins, the binding cavity in iLOV had a considerably higher average B-factor (*B_avg_* = 23.29) relative to the binding cavity in EcFbFP (*B_avg_* = 17.18). B-factor values (also known as temperature factor) represent the extent of disorder associated with the spatial locations of amino acids in protein structures. High values of B-factor indicate uncertainty in precisely locating the coordinates of amino acid side chains in the crystal structure of a protein, which is usually the case for side chains that are relatively less restrained, such as surface exposed residues. In particular, protein engineering techniques that specifically target high B-factor residues for mutations (relative to lower B-factor amino acids) have been shown to improve stability of enzymes [40], [41]. In the context of EcFbFP and iLOV, a higher B-factor in the iLOV binding site may indicate a binding cavity that is less rigid relative to the chromophore-binding cavity in EcFbFP. For example, an arginine residue at position 58 in iLOV, which is directly involved in hydrogen bonding with FMN, has a substantially large B-value of 46.61. The corresponding arginine in EcFbFP (R79) has a B-value of 17.75. The significantly larger B-value associated with a key residue involved in hydrogen bonding with FMN ring may constitute a structural basis for the lower holoprotein fraction in iLOV.

**Table 2:** Fraction of fluorescent holoprotein.

Protein	*F_holo_*
PpFbFP	0.60±0.02
EcFbFP	0.68±0.02
iLOV	0.39±0.01

The fraction of FbFP that exists in an FMN-bound holo form (*F_holo_*) is calculated based on absorption at 450 nm by the holoprotein.

### Oligomeric State of FbFPs

Monomeric fluorescent reporter proteins are desirable for generating translational fusions with minimal imprint. Multimeric fluorescent proteins often impair the folding and functionality of proteins to which they are translationally fused. Furthermore, for applications involving Förster resonance energy transfer (FRET), oligomerization of fluorescent reporters can significantly misconstrue interpretation of protein-protein interactions. FbFPs exhibit an overall small size (≈130 amino acids), which provides a considerable advantage over the bulkier GFP-family proteins (≈240 amino acids) in constructing translational fusions with small imprint. To this end, we characterized the oligomeric states of these proteins using size exclusion chromatography (**Table 3**). Using this approach, we determined that iLOV is a monomer, whereas EcFbFP and PpFbFP are predominantly dimers (**Figure S1**). We found that the oligomeric state of FbFPs was relatively insensitive to ionic strength, and the oligomeric states remained intact in both high salt (1 M and 0.5 M NaCl) and lower salt concentrations (150 mM NaCl). iLOV is the smallest protein in the FbFP family, consisting of only 110 amino acids. Therefore, based on its size and monomeric structure, iLOV has an exceedingly small imprint as a fluorescent tag.

**Table 3:** Oligomeric state of FbFPs.

Protein	0.15 M NaCl	0.5 MNaCl	1 M NaCl	Oligo state	Mol. mass(kDa)
PpFbFP	1.9	1.7	1.5	dimer	32.4
EcFbFP	1.9	1.7	1.6	dimer	30
iLOV	0.9	0.7	0.8	monomer	12

Oligomeric states of FbFPs were assessed in buffers of varying ionic strengths using size exclusion chromatography. Values represent the mean of duplicate experiments. Ionic strength was regulated by adjusting the concentration of NaCl in the various buffers.

### Effect of pH on FbFP Fluorescence

**Figure 3:**
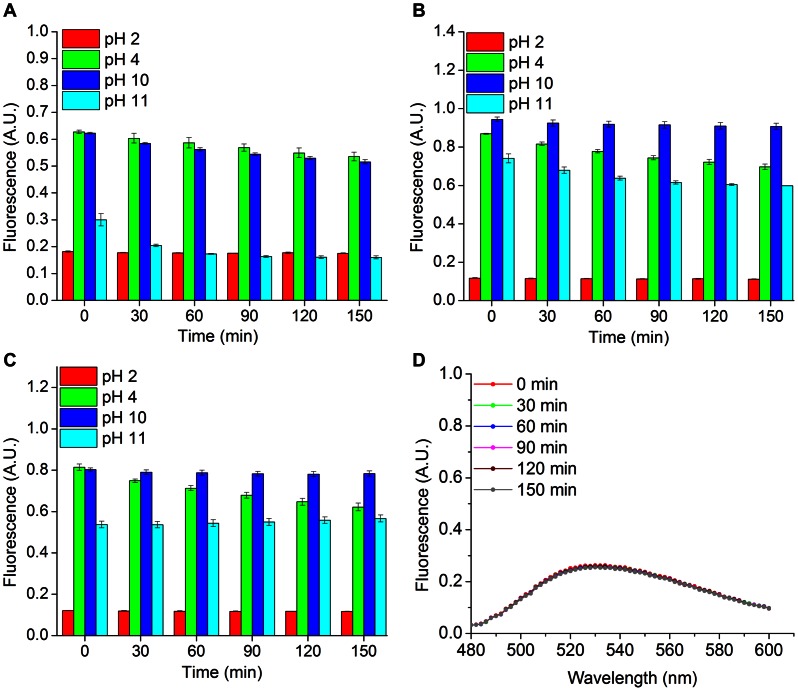
Effect of pH on FbFP fluorescence. Histograms depict the fraction of peak fluorescence (measured at 495 nm at pH 7 for PpFbFP and EcFbFP and pH 6 for iLOV) retained by A) PpFbFP, B) EcFbFP, and C) iLOV after incubation in buffers of pH 2, 4, 10, and 11 for 2.5 h. D) FbFPs are readily denatured by incubation at pH 2 and are characterized by the appearance of a flavin-spectrum with a peak at 525 nm, as is depicted for iLOV.

Fluorescent proteins with a broad functional pH range are desirable for intracellular imaging in alkaliphilic life forms and for studying acidic cellular environments such as endosomes, lysosomes, and plant vacuoles. We characterized the pH sensitivities of FbFPs by incubating purified fractions of FbFPs in buffers of pH 2, 4, 10, and 11 for several hours. We verified that PpFbFP and EcFbFP fluoresce maximally at pH 7, while iLOV has maximum fluorescence emission at pH 6 (**Figure S2**). Therefore, we normalized peak fluorescence emission intensity (measured at 495 nm) at a particular pH to the maximum fluorescence emission intensity measured at pH 7 (PpFbFP and EcFbFP) or pH 6 (iLOV) (**Figure 3**). Changes in overall quantum yields of FbFPs at different pH values are provided as supporting information (**Text S3**). We found that both EcFbFP and iLOV exhibit fluorescence over a broad pH range (pH 4 to 11), generally retaining 60–70% of their maximum fluorescence up on prolonged incubation at pH 4. Furthermore, iLOV and EcFbFP strongly fluoresce in highly alkaline conditions and retain nearly 60% of their maximum fluorescence when incubated at pH 11 for over two hours. Moreover, PpFbFP is stable at pH 4 (53% of maximum fluorescence), but readily loses fluorescence at pH 11. Finally, all three FbFPs rapidly lose fluorescence at very low pH values, conditions under which the fluorescence emission peak at 495 nm disappears and the FMN-specific emission spectrum begins to emerge (**Figure 3**, **Figure S3**). In contrast to FbFPs, YFP has a pK_a_ of 5.5–6.5 [42] and readily loses fluorescence in acidic conditions, retaining less than 10% of its maximum fluorescence up on incubation at pH 4 (**Figure S4**).

### Thermal Stability of FbFPs

**Figure 4:**
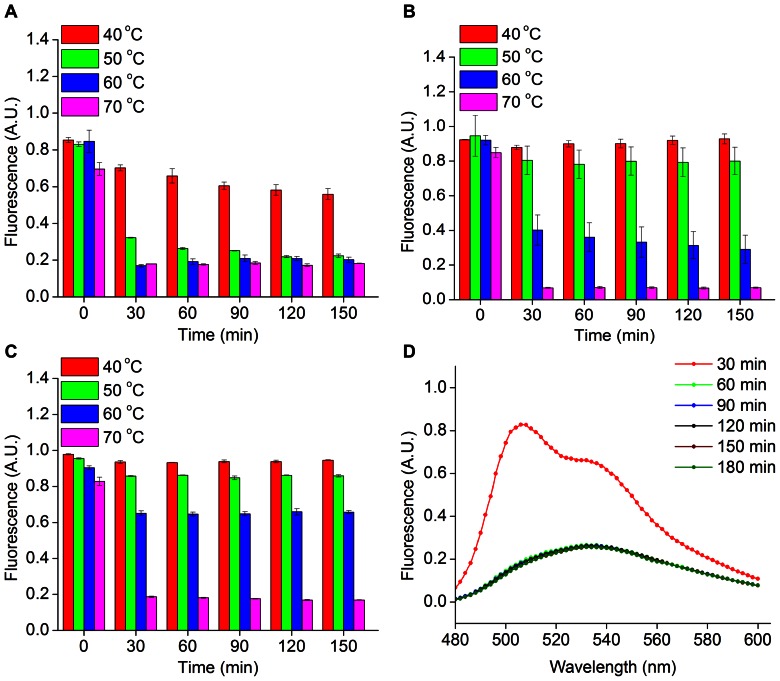
Effect of temperature on FbFP fluorescence. Histograms depict the fraction of peak fluorescence (measured at 495 nm at room temperature) retained by A) PpFbFP, B) EcFbFP, and C) iLOV after incubation at 40°C, 50°C, 60°C, and 70°C for 2.5 h. D) FbFPs are readily denatured at 70°C and are characterized by the appearance of a flavin-spectrum with a peak at 525 nm, as is depicted for iLOV.

Thermophilic microorganisms are promising host platforms for several bioprocess and biotechnology applications. Thermostable fluorescent proteins extend the scope of fluorescent reporters to investigate biological processes in thermophiles. Wild type GFP matures very slowly at temperatures exceeding 20°C; however, several folding mutations have been identified to improve the thermal tolerance of GFP-based proteins. In this way, GFP mutants have been optimized for fluorescence at elevated temperatures up to 80°C [43], [44] (**Figure S4**). However, oxygen-dependent maturation of fluorescence in GFP-based reporters precludes the application of thermostable GFP variants to vast majority of thermophilic microbes, which are commonly obligate anaerobes. In order to assess the ability of FbFPs to function in high temperature conditions, we determined the temperature dependence of FbFP fluorescence using purified protein preparations (**Figure 4**). Specifically, we determined peak fluorescence emission intensity (measured at 495 nm) at a particular temperature and normalized this value to the peak fluorescence emission intensity measured at room temperature (25°C). Changes in overall quantum yields of FbFPs at different temperatures are provided as supporting information (**Text S3**). We found that iLOV significantly outperforms PpFbFP and EcFbFP at elevated temperatures and retains more than 80% and 60% of its room temperature fluorescence upon prolonged incubation at 50°C and 60°C, respectively. Moreover, EcFbFP retains approximately 80% of its room temperature fluorescence upon incubation at 50°C. However, higher temperatures lead to rapid loss of fluorescence in EcFbFP. In all cases, continued incubation of FbFPs at 70°C leads to protein precipitation along with gradual reduction of the 495 nm emission peak and the appearance of a smoother emission spectrum with a peak at 525 nm, which is characteristic of free FMN (**Figure 4**, **Figure S5**).

### Fluorescence Recovery after Denaturation

**Figure 5:**
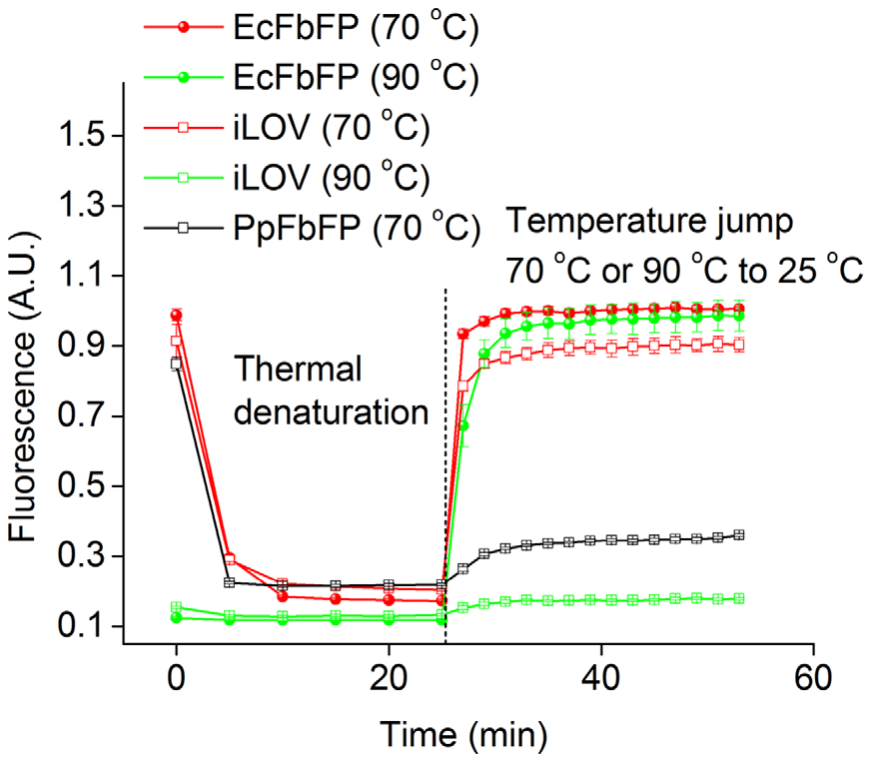
Fluorescence recovery after denaturation of FbFPs. FbFPs were denatured by incubation at 70 or 90°C for 25 minutes. Denaturation at 70°C was accompanied by the progressive loss of peak fluorescence emission at 495 nm. However, at 90°C, denaturation was almost immediate, as is indicated by the low values of fluorescence emission at the initial time point for iLOV and EcFbFP denatured at 90°C. At t = 25 minutes, the temperature was rapidly (∼2–3 minutes) lowered from 70 or 90°C to 25°C using a temperature controlled fluorescence spectrometer. EcFbFP and iLOV rapidly regained fluorescence following thermal denaturation 70°C indicating fast renaturation and fluorescence recovery. Fluorescence recovery was observed in EcFbFP upon thermal denaturation at 90°C, as well. In both cases, PpFbFP was irreversibly denatured upon thermal treatment.

Maturation of fluorescence in GFP is mediated by oxidation of the cyclic tripeptide chromophore, which requires more than an hour at room temperature [10]. Despite the development of fast-maturing GFP variants such as venus [45], chromophore oxidation and maturation of fluorescence requires nearly 10–40 minutes for recovery of half maximal fluorescence even in the fastest maturing variants [42]. This constitutes a major bottleneck in applying GFP to study dynamic biological events. Fluorescence maturation kinetics is typically monitored by observing the recovery of fluorescence emission following renaturation of denatured and sodium dithionite-reduced GFP preparations. We adopted an analogous approach to investigate the maturation of fluorescence in FbFPs by first denaturing FbFPs at high temperature, followed by rapid cooling of the proteins to room temperature (**Figure 5**). Protein unfolding and refolding were monitored using fluorescence spectroscopy, where we used the distinct signatures of the fluorescence emission spectra of the FbFP holoprotein and free FMN to distinguish apo-protein from intact FbFP holoprotein [46]. First, FbFPs were readily denatured by heating at 90°C for 25 minutes, whereupon we observed that the unfolded proteins aggregated into visible precipitates. In the case of EcFbFP, fluorescence was readily restored upon rapid cooling of samples back to room temperature (**Figure 5** **, Figure S6A**). However, PpFbFP and iLOV were irreversibly denatured after the high temperature cycle at 90°C, and cooling failed to restore fluorescence for these proteins. On the other hand, reversible denaturation of iLOV could be achieved by thermal denaturation at a lower temperature of 70°C instead of 90°C (**Figure 5**). For EcFbFP and iLOV, recovery of fluorescence was rapid and appeared to occur within less than 2–3 minutes, which is approximately equal to the time required by the temperature controlled fluorescence spectrometer to cool from 90°C (or 70°C) to room temperature (25°C). In contrast, thermally denatured YFP recovered ≈46% of its fluorescence in the same time in which EcFbFP fluorescence was restored to greater than 90% (**Figure S6B**). Furthermore, we note that apparent rate of fluorescence recovery in YFP in this experiment is substantially faster than the actual rate of natural fluorescence maturation because the functional YFP chromophore is not chemically reduced in the course of thermal denaturation. In reality, acquisition of fluorescence in YFP involves cyclization of the tripeptide chromophore followed by oxidation, which substantially slows the overall kinetics of fluorescence maturation.

### Effect of Strongly Reducing Conditions on FbFP Fluorescence

The GFP chromophore is readily quenched by high concentrations of strong reductants such as sodium dithionite [12]. As a result, fluorescence in GFP-based proteins is sensitive to reducing conditions, which precludes the use of GFP-based reporters in highly reducing environments as are typically encountered during the cultivation of several obligate anaerobes such as methanogens. We investigated the sensitivity of FbFP fluorescence to reducing conditions by treating purified FbFPs with sodium dithionite at ≈1000-fold excess molar concentrations (**Figure S7**). Under these conditions, the reduction potential of the solution is ≈−660 mV, which establishes a very strong reducing environment. In this experiment, we recorded fluorescence emission over a period of 3 hours. We determined that fluorescence emission from all three FbFPs was insensitive to reducing conditions, which could in part reflect the general insensitivity of the FMN chromophore to reductants such as sodium dithionite or dithiothreitol in aerated conditions [46]. It is noteworthy that under similar conditions, the GFP chromophore has been reported to be readily reduced to a nonfluorescent form [42], [45].

### FbFPs as Real Time Reporters of Gene Expression in E. coli

Fluorescent reporters are widely used to quantify gene expression by constructing transcriptional fusions between the reporter and a promoter of interest. Experimental estimates of promoter activity can be integrated with mathematical modeling to discern regulatory mechanisms underlying genetic networks, as well as to investigate the response of a gene network to environmental stimuli [47], [48]. In order to investigate the suitability of FbFPs as reporters of gene expression in wild type *E. coli* MG1655, we generated a series of transcriptional reporter constructs based on FbFPs. Specifically, we cloned and expressed the FbFPs in *E. coli* under transcriptional control of a T5-lacO hybrid bacteriophage promoter, whose activity can be induced with isopropyl β-D-1-thiogalactopyranoside (IPTG) [49]. Cells were cultured in minimal M9 medium supplemented with glucose or glycerol as the carbon source and promoter activity was monitored using fluorescence spectrometry. Cellular metabolic burden upon expression of fluorescent reporter proteins may affect stable labeling with any fluorescent probe. To this end, we sought to determine whether FbFP overexpression could burden cells owing to its affinity for FMN, which is a key cellular metabolite.

**Figure 6:**
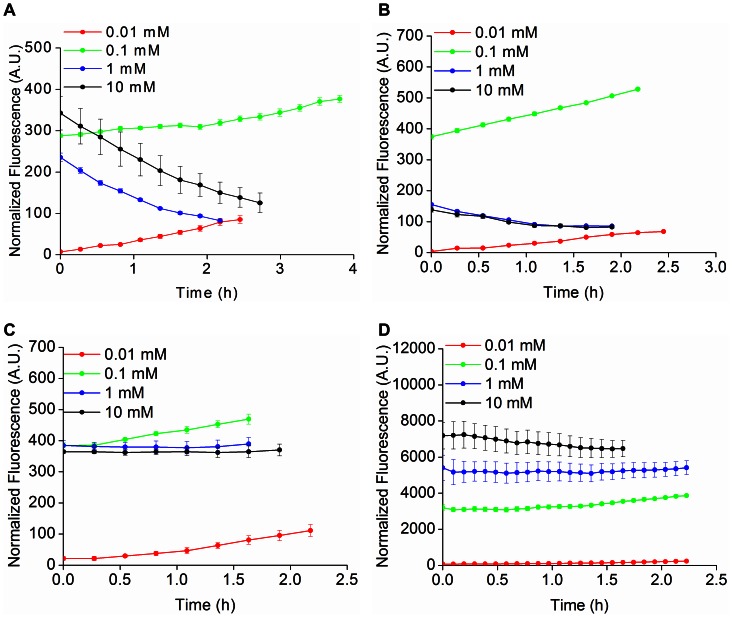
FbFPs as transcriptional reporters of T5 promoter activity in *E.coli* grown in M9-glucose. FbFPs and YFP were expressed in *E. coli* MG1655 cells using an IPTG-inducible T5 promoter. Fluorescence and optical density were recorded over the logarithmic phase of cell growth (typically after ≈2 hours of lag phase following re-inoculation of overnight culture) in M9 medium supplemented with glucose at 20 mM concentration as the carbon source. IPTG concentrations were varied to span different levels of transcriptional activity of the T5 promoter. Fluorescence was divided by the optical density at 600 nm. Normalized fluorescence values are depicted for A) PpFbFP, B) EcFbFP, C) iLOV, and D) YFP. Steady state promoter activity was verified using YFP as a reporter. PpFbFP and EcFbFP deviated considerably from the expected steady state promoter dynamics. However, iLOV revealed close agreement with the YFP expression profile over a broad range of IPTG concentrations. Promoter activities are depicted corresponding only to the logarithmic phase of cell growth (0.4< *A_600nm_* <0.8). As the duration of the logarithmic phase varies for *E. coli* cells expressing distinct transcriptional reporter constructs, so does the time frame over which the promoter activity is depicted in the figures.

**Figure 7:**
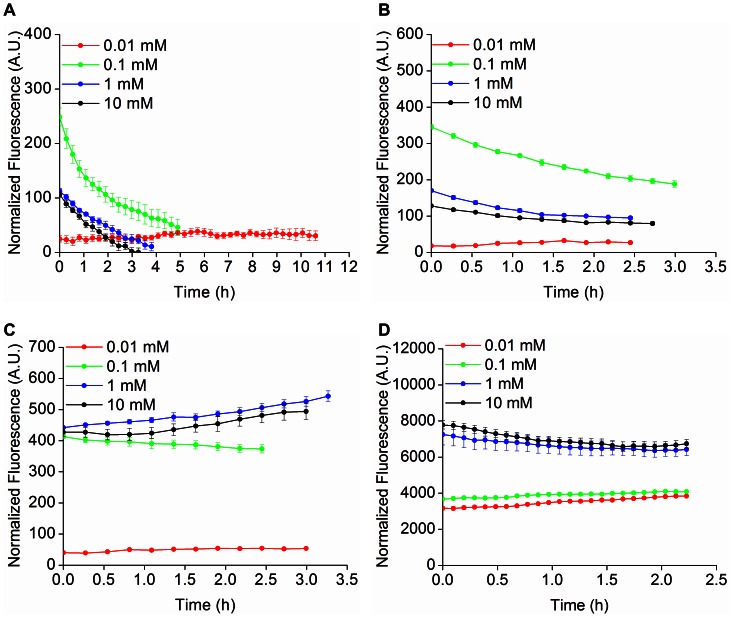
FbFPs as transcriptional reporters of T5 promoter activity in *E.coli* grown in M9-glycerol. FbFPs and YFP were expressed in *E. coli* MG1655 cells using an IPTG-inducible T5 promoter. Fluorescence and optical density were recorded over the logarithmic phase of cell growth (typically after ≈2 hours of lag phase following re-inoculation of overnight culture) in M9 medium supplemented with glycerol at 0.5% (v/v) concentration as the carbon source. IPTG concentrations were varied to span different levels of transcriptional activity of the T5 promoter. Fluorescence was divided by the optical density at 600 nm. Normalized fluorescence values are depicted for A) PpFbFP, B) EcFbFP, C) iLOV, and D) YFP. Steady state promoter activity was verified using YFP as a reporter. PpFbFP and EcFbFP deviated considerably from the expected steady state promoter dynamics. However, iLOV revealed close agreement with the YFP expression profile over a broad range of IPTG concentrations. Promoter activities are depicted corresponding only to the logarithmic phase of cell growth (0.4< *A_600nm_* <0.8). As the duration of the logarithmic phase varies for *E. coli* cells expressing distinct transcriptional reporter constructs, so does the time frame over which the promoter activity is depicted in the figures.

In these experiments, we compared the growth rates of cells expressing the T5-FbFP transcriptional fusions to that of cells expressing YFP during growth in M9-glucose and M9-glycerol. In addition to growth rates, we simultaneously monitored promoter activities using fluorescence. IPTG concentrations were varied (0.01, 0.1, 1, and 10 mM) to access different levels of transcriptional activity. Whole cell fluorescence and absorbance at 600 nm (*A_600nm_*) were measured (**Figures S8–10**, **Materials and Methods**). Analysis of promoter activity was restricted to the exponential phase of cell growth (0.4< *A_600nm_* <0.8). During exponential phase growth, intracellular synthesis of fresh reporter protein is balanced by dilution due to cell growth, presumably resulting in steady-state promoter dynamics, characterized by relatively small changes in promoter activity [50], [51]. Therefore, fluorescence from reporters coupled to the T5 promoter is expected to remain invariant during the exponential growth phase. We verified that fluorescence from YFP coupled to the T5 promoter indeed remains nearly constant over the logarithmic growth phase of cells (**Figures 6D** **,** **7D**). In addition, steady state promoter dynamics was further validated using Western blot measurements to estimate the production of YFP at three time points corresponding to exponential phase of growth (**Figure S11**). In the case of transcriptional fusions between FbFPs and the T5 promoter, only iLOV accurately captured the expected steady state dynamics of the T5 promoter and revealed good agreement with the YFP data (**Figures 6** **–** **7** **,S9–S10**) over a wide range of IPTG concentrations. In contrast, EcFbFP and PpFbFP fluorescence revealed a steadily decreasing trend in *E. coli*, particularly during growth in glycerol (**Figure 7**). Furthermore, enhancing the ClpXP protease dependent *in vivo* degradation rates of EcFbFP (and iLOV) through the construction of LVA-tagged variants did not substantially alter the fluorescence profiles (**Figure S12**). Based on Western blot experiments, the decreasing fluorescence observed in the case of PpFbFP and EcFbFP can be explained in part by intracellular degradation of these proteins upon overexpression (**Figure S11**). Progressive loss of fluorescence with time may also be a consequence of incomplete formation of PpFbFP and EcFbFP holoproteins, if the rate of freshly synthesized cellular proteins far exceeds the available intracellular FMN pool, particularly in the case of cells grown using a poor carbon source such as glycerol. Several intracellular mechanisms can explain the differences in expression profiles between iLOV and the bacterial FbFPs. For example, expression of PpFbFP and EcFbFP may impose a greater burden on cellular flavin resources owing to their dimeric nature or stronger affinity for FMN compared to iLOV (**Table 2**). As a result, progressive depletion of cellular flavin would lead to *in vivo* synthesis of EcFbFP and PpFbFP existing in predominantly apo-forms. In this scenario, the decreasing fluorescence trend would be the result of dilution by cell growth of previously synthesized holo-EcFbFP and PpFbFP.

Finally, regarding cell doubling times, we observed that EcFbFP and iLOV expressing cells showed comparable exponential-phase growth rates as YFP expressing cells in glucose and glycerol-based media (**Table 4**). However, we observed retardation in the log-phase growth rates of PpFbFP expressing cells compared to cells expressing YFP, in both glucose and glycerol-based growth media (**Table 4**). Our results suggest that expression of EcFbFP and iLOV impact cellular growth in a manner analogous to YFP or GFP, whereas PpFbFP expression clearly affects cellular growth rates, which could arise due to an additional metabolic burden of PpFbFP on *E. coli* cell growth.

**Table 4:** Doubling times of *E. coli* MG1655 expressing FbFPs, GFP, and YFP.

Expressionconditions	PpFbFP	EcFbFP	iLOV	YFP (GFP)
P_T5-_glucose(0.1 mM IPTG)	3.81±.04	2.36±.02	2.01±.01	2.20±.15
P_T5-_glucose(1 mM IPTG)	2.46±.06	1.76±.03	2.04±.02	2.20±.03
P_T5-_glucose(10 mM IPTG)	2.88±.04	2.00±.08	2.17±.14	2.19±.09
P_T5-_glycerol(0.1 mM IPTG)	5.32±.38	2.78±.04	2.29±.04	3.57±.03
P_T5-_glycerol(1 mM IPTG)	5.72±.18	2.41±.05	3.17±.04	3.39±.13
P_T5-_glycerol(10 mM IPTG)	4.72±.20	3.99±.67	3.61±.19	3.33±.06
P_λ-_glucose(constitutive)	n.d.	n.d.	1.95±.14	1.70±.04
P_λ-_glycerol(constitutive)	n.d.	n.d.	2.38±.07	2.89±.22

PpFbFP, EcFbFP, iLOV, and YFP were expressed in *E. coli* MG1655 using an IPTG-inducible phage T5 promoter harbored in a medium copy plasmid (pQE80L). In addition, iLOV and GFP were expressed using a constitutive phage lamda promoter in a low copy plasmid (pAM06-tet). Cells were grown in M9 media using glycerol or glucose as the carbon source and doubling times were estimated over the logarithmic phase of cell growth corresponding to 0.4< *A_600nm_* <0.8.

### FbFPs as Real Time Reporters: Constitutive Promoters & Anaerobic Gene Expression

**Figure 8:**
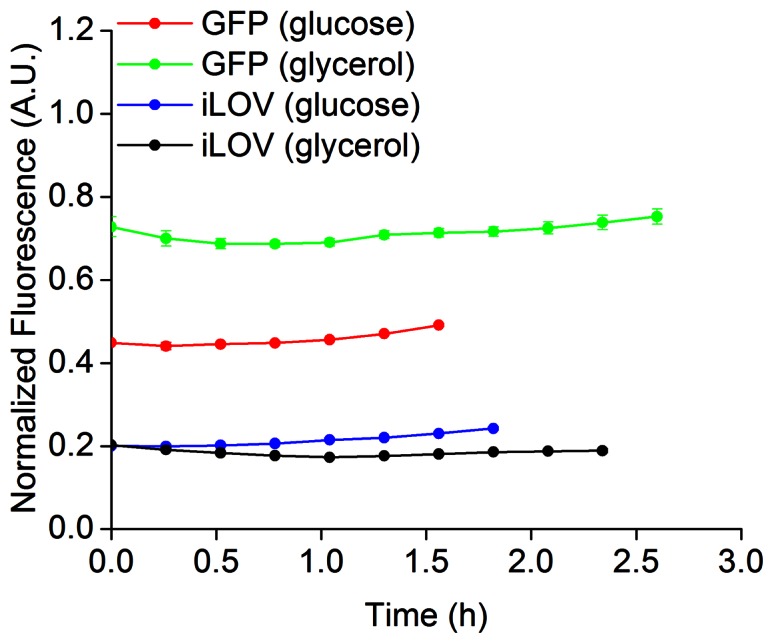
iLOV as a reporter of phage lambda promoter activity in *E. *coli. GFP and iLOV were expressed in *E. coli* MG1655 cells using a constitutive phage lambda promoter. Fluorescence and optical density were recorded over the logarithmic phase of cell growth (typically after ≈2 hours of lag phase following re-inoculation of overnight culture) in M9 medium supplemented with glucose at 20 mM or glycerol at 0.5% as the carbon source. Fluorescence was divided by the optical density at 600 nm and normalized to the maximum value reached over 16 h. of cell growth. Steady state promoter activity was verified using GFP as a reporter. We generally observed good agreement between the iLOV and GFP expression profiles. Promoter activities are shown corresponding only to the logarithmic phase of cell growth (0.4< *A_600nm_* <0.8). As the duration of the logarithmic phase varies for *E. coli* cells expressing the GFP and iLOV reporter constructs, so does the time frame over which the phage λ promoter activity is depicted in the figures.

**Figure 9:**
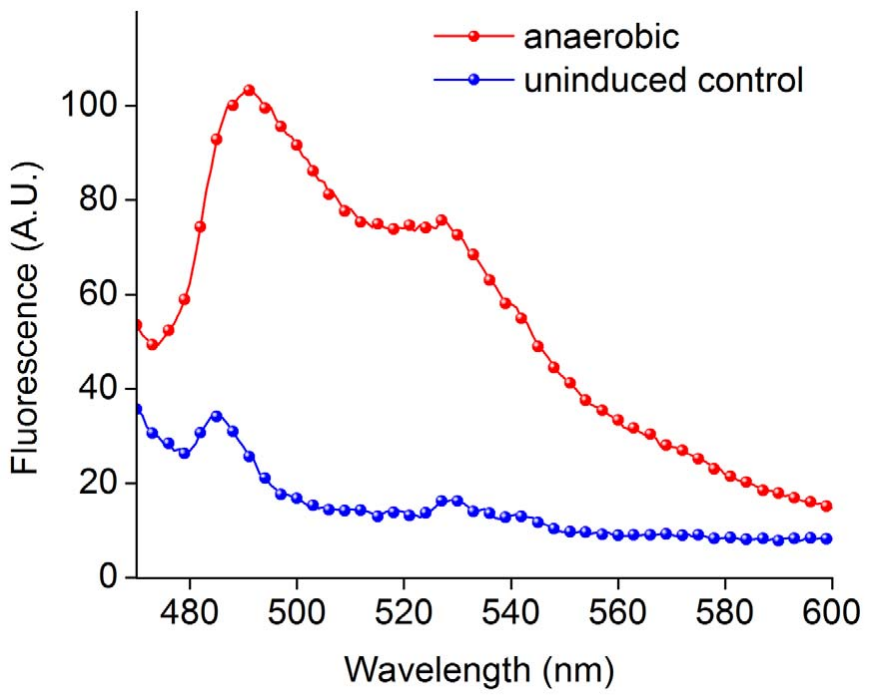
Expression of iLOV in anaerobically cultured *E. coli* MG1655. *E. coli* MG1655 cells were grown in M9 medium supplemented with 20 mM glucose (carbon source) and 20 mM potassium nitrate (electron acceptor). Anaerobic conditions were established by growing the cells in air-tight stoppered Balch tubes filled completely with growth media and evacuated for 30 minutes using vacuum suction. Anaerobic cultures were grown without shaking. Cells were induced with 0.5 mM IPTG for 12 hours, resuspended in phosphate buffered saline and scanned using fluorescence spectrometry (470 nm excitation).

In order to further evaluate the scope of iLOV as a robust reporter of gene expression, we constructed a transcriptional fusion between iLOV and a constitutive phage lambda P_L_ promoter [52]. The promoter-reporter cassette was integrated in a low copy promoter probe plasmid constructed in our lab (**Materials and Methods**). The phage lambda promoter contains operator sequences recognized by the tetracycline repressor TetR protein. In the absence of TetR in *E. coli* MG1655, the promoter behaves as a constitutive promoter. *E. coli* cells harboring the above expression constructs were grown in M9 medium using glucose or glycerol as the carbon source. We compared gene expression profiles in the logarithmic phase of cell growth using iLOV and GFP as reporters. Promoter activity measurements and growth curves over the entire cell growth period are provided as supporting information (**Figure S13**). Similar to results obtained with the T5 promoter, we observed good agreement between gene expression assayed using iLOV and GFP (**Figure 8**). Furthermore, growth rates were also comparable in the case of *E. coli* cells expressing iLOV and GFP from the phage lambda promoter (**Table 4**). Overall, iLOV significantly outperforms EcFbFP and PpFbFP as a real-time reporter of transcriptional activity in *E. coli*. Finally, we verified cellular expression and fluorescence of iLOV under anaerobic conditions of *E. coli* culture (**Figure 9**). Previously, anaerobic expression of PpFbFP and EcFbFP was demonstrated by Drepper and colleagues, as well as in our lab [19], [30], [38]. However, this work presents the first demonstration of “anaerobic” fluorescence using iLOV as a molecular reporter. Existing options for fluorescent labeling of anaerobic biosystems are typically limited to small molecule based probes, such as fluorescein and rhodamine-conjugated bi-arsenical dyes (FlAsH and ReAsH) and O^6^-alkylguanine transferase and haloalkane dehydrogenase-based enzymatic protein tags (SNAP and HALO tags) [53]–[56]. Small molecule-based approaches enable fluorescence imaging using probes that are typically several-fold brighter than iLOV. For example, SNAP tags based on ATTO 488 (brightness = 72,000) and ATTO 590 (brightness = 96,000) dyes are 16–20 fold brighter than iLOV. Despite the improved signal-to-noise ratios achievable with small molecule based probes, their application is frequently limited by poor permeability and cellular toxicity of the organic dyes. From this perspective, FbFPs in general and iLOV in particular constitute a class of small molecule based fluorescent probes that circumvent the aforementioned issues by binding flavin, which is a nontoxic metabolite endogenous to most cells. Taken together with its strong suitability as a transcriptional reporter, iLOV emerges as a promising reporter of gene expression with potential application in obligate and facultative anaerobes.

### Conclusions

In this work, we demonstrate that FbFPs represent an emerging class of fluorescent reporters with several useful characteristics for application in a broad range of biological systems. Previous applications of FbFPs have predominantly focused on imaging in hypoxic and anoxic environments. However, in this work, we demonstrate that in addition to oxygen-independent fluorescence, FbFPs integrate several key properties that render them immensely useful as biological probes. In particular their small size (≈55% the size of GFP), broad pH range, thermal, and redox tolerance make them robust reporters with a potential to address several biological systems of outstanding importance that are poorly suited to investigation using GFP. For example, fluorescent reporter proteins are routinely employed for on-line detection of recombinant protein production in order to identify optimal bioprocess parameters to ensure maximum productivity. The use of GFP and analogous reporters in such conditions is rendered difficult by the incomplete and slow maturation of the GFP-chromophore as semi-anaerobic to anaerobic conditions often prevail in bioprocess production platforms (*e.g*., fermentation) [57]. In addition, the large size of GFP has been shown to frequently interfere with the folding of partner fusion proteins, *e.g.*, in the case of secreted proteins [16]. FbFPs exhibit an overall small size (**Table 3**), robust folding (**Figure 5**), and oxygen-independent fluorescence, all of which makes these probes function as superior fluorescent reporters under these conditions. Strikingly, rapid and robust refolding of FbFPs (specifically, iLOV and EcFbFP) in 2–3 minutes following thermal denaturation is suggestive of fast maturation of fluorescence in this class of proteins (**Figure 5**). This constitutes a significant advantage over GFP-based probes, because even the fastest maturing GFP variants (notably, venus and Tag-GFP2) require 10–40 minutes for maturation of half-maximal fluorescence [42], [45], [58]. As fast maturing reporters, FbFPs may be immensely advantageous for precisely resolved temporal studies such as monitoring short-lived proteins and early detection of promoter activation. Moreover, although the intrinsic pH-sensitivity of the GFP chromophore has been exploited to design pH-responsive probes [59], the rapid loss of GFP fluorescence at low pH complicates fluorescence measurements of acidic biological processes such as endocytosis, synaptic vesicle fusion, and periplasmic or extracellular protein secretion. In this sense, the broad pH tolerance of iLOV and EcFbFP, spanning pH 4–11 (**Figure 3**), makes them ideally suited to monitor such physiological events. Based on our results, we identify iLOV as the most promising member in the FbFP family with its substantially smaller size (110 amino acids, monomeric) as well as robust fluorescence under extremes of pH and temperature (retaining ≈60% of maximum fluorescence at pH 4 and 11, and at 60°C). Importantly, iLOV emerges as a suitable noninvasive real-time reporter of transcriptional activities of bacteriophage promoters (**Figures 6** **–** **8**). As iLOV fluorescence is detectable even under anaerobic conditions (**Figure 9**), it can serve as a valuable probe for promoter activity under anaerobic conditions. As an example, there is considerable interest in the development of promoters that are tunably regulated by environmental cues commonly encountered in industrial-scale biotechnological applications such as oxygen depletion and the onset of anaerobiosis [60]. The application of GFP for screening oxygen-induced promoters requires post-incubation of anaerobically cultivated cells in well aerated conditions to initiate chromophore oxidation and maturation in GFP [61]. FbFP-based reporters such as iLOV can substantially ease and intensify the development of such promoter cassettes. Finally, the limited brightness (product of quantum yield and molar extinction coefficient) of FbFPs is currently a major bottleneck restricting their versatile and widespread utilization as practical reporters. Indeed, the brightest FbFPs (iLOV and EcFbFP, brightness = 4250) are only 7–12% as bright as spectrally similar GFP-based probes such as mTFP1 (brightness = 54,400), EGFP (brightness = 33,600), and Venus (brightness = 52,554). Therefore, future work at developing FbFP-based fluorescent reporters should focus on enhancing brightness through improvements in fluorescence quantum yield or tighter binding with the flavin chromophore [38]. Overall, we expect this study to furnish an important characterization framework to encourage the continued development of FbFPs as well as encourage their application to fundamental and applied biological studies.

## Methods

### Bacterial Strains and Growth Media

E. coli DH5α cells were routinely used for cloning and propagation of the wild type FMN-based fluorescent protein (FbFP) genes from Pseudomonas putida, Bacillus subtilis, and Arabidopsis thaliana. E. coli BLR (DE3) expression strains (EMD Chemicals) were used for protein expression. Transcriptional assays by fluorescence spectroscopy were performed using wild type E. coli MG1655 cells. Cells were grown with vigorous shaking (200 r.p.m.) at 37°C in Lennox broth (10 g/L tryptone, 5 g/L yeast extract, 5 g/L NaCl) or on solid 1.5% agar plates containing Lennox medium and incubated at 37°C for 20 hours. Ampicillin at a concentration of 100 µg/mL was used for maintenance of the pQE80L plasmids. Kanamycin at a concentration of 30 µg/mL was used for maintenance of the pAM06-tet and pAM09-tet plasmids. For gene expression experiments, cells were grown in M9 minimal medium (12.8 g/L Na_2_HPO4.7H2O, 3 g/L KH_2_PO_4_, 0.5 g/L NaCl, and 1 g/L NH4Cl) supplemented with 0.1% casamino acids and 0.5% glycerol or 20 mM glucose as the carbon source. For anaerobic cultivation, M9-glucose medium was supplemented with 20 mM potassium nitrate as the electron acceptor. Anaerobic culture conditions were established by growing cells in air-tight stoppered Balch tubes completely filled with growth media and evacuated using vacuum suction for 30 minutes. Anaerobic cultures were grown without shaking. Lennox broth, ampicillin, casamino acids, and agar were purchased was purchased from Fisher Scientific (Pittsburgh, PA). All other chemicals were purchased from Sigma-Aldrich (St. Louis, MO).

### Cloning of FbFP Genes

FbFP genes were synthesized by GenScript (Piscataway, NJ) or Integrated DNA Technologies (Coralville, IA) and the sequences are provided as supporting information (**Text S4**). The gene for the yellow fluorescent protein (YFP) was amplified from a CRIM promoter probe plasmid [62], which was a kind gift from Prof. Christopher Rao (University of Illinois at Urbana-Champaign). The genes were cloned in the pQE80L expression vector from Qiagen (Valencia, CA) using *BamHI* and *HindIII* restriction enzymes. The constructs were respectively designated pQE80L-PpFbFP, pQE80L-EcFbFP, pQE80L-iLOV, and pQE80L-YFP. PCR amplification, restriction digestion, and ligation were accomplished using standard protocols [63]. Briefly, PCR was carried out in 50 µL reaction volume using 10 ng of template DNA and 0.5 µM primers, 0.2 mM dNTPs and 2.5 units Taq DNA polymerase. The PCR cycle consisted of an initial denaturation at 94°C for 2 minutes followed by 25 cycles of 94°C for 30 s, 55°C for 30 s and 72°C for 45 s (1 minute for YFP). A final extension step at 72°C for 10 minutes was employed to complete synthesis of full-length templates. Amplicons were digested with 10 units each of *BamHI* and *HindIII* restriction endonucleases at 37°C for 1 hour, purified by gel electrophoresis and subsequently ligated into pQE80L expression vector digested with *BamHI* and *HindIII* using similar reaction conditions. Ligation reactions were performed using 400 units T4 DNA ligase in a 20 µL reaction volume at room temperature for 1 h. Promoter probe plasmids pAM06-tet and pAM09-tet were constructed based on a previously reporter pUA139 promoter probe vector that expresses GFP cycle 3 mutant under the control of an upstream promoter of choice [50]. We engineered the plasmid to include a 3-frame stop codon and a strong synthetic ribosome binding site between the GFP gene and the upstream promoter. In order to make the promoter probe vector modular, we incorporated *NheI* and *HindIII* restriction sites flanking the GFP reporter gene. The ribosome binding site was flanked by *KpnI* and *NheI* restriction sequences. We constructed a synthetic phage lambda P_L_-tetO hybrid promoter [52] by annealing complimentary oligonucleotides. The promoter was ligated in the plasmid construct using *BamHI* and *XhoI* restriction enzymes and T4 DNA ligase. An rrnB2 transcriptional terminator was included upstream of the promoter to prevent divergent transcription. The modified promoter probe plasmid was designated as pAM06-tet. A promoter probe plasmid harboring iLOV was constructed by digesting pAM06-tet with *NheI* and *HindIII* and ligating iLOV, which had been digested with the same enzymes. This construct was designated pAM09-tet. All plasmid constructs were propagated by transformation in *E. coli* DH5α cells using heat shock at 42°C. Cells were plated on LB-agar supplemented with ampicillin or kanamycin for selection. Plasmids were isolated from *E. coli* DH5α transformants (Qiagen Miniprep kit) and used to transform *E. coli* BLR (DE3) cells for protein expression or *E. coli* MG1655 cells for gene expression studies. Plasmid constructs pQE80L-PpFbFP, pQE80L-EcFbFP, pQE80L-iLOV, and pQE80L-YFP were employed for protein expression as well as promoter activity measurements of the IPTG-inducible phage T5 promoter. Plasmid constructs pAM06-tet and pAM09-tet were utilized for assaying promoter activity of the constitutive phage lambda promoter. **Table 5** contains a list of oligonucleotide sequences corresponding to the primers, phage lambda promoter, synthetic ribosome binding site, and transcriptional terminator. Restriction enzymes were purchased from New England Biolabs (Ipswich, MA), and primers were synthesized by Integrated DNA Technologies (Coralville, IA).

**Table 5:** List of oligonucleotides and plasmid constructs used in this work.

S.No.	Oligonucleotide	Sequence
1	PpFbFP_fwd_BamHI	GGATCC ATGATCAACGCAAAACTCCTG
2	PpFbFP_rev_HindIII	AAGCTT TCAGTGCTTGGCCTGGCC
3	EcFbFP_fwd_BamHI	GTTTCTTCGGATCCATGGCGTCGTTCCAGTCGTT
4	EcFbFP_rev_HindIII	GTTTCTTCAAGCTTTTACTCGAGCAGCTTTTCATATTCCTTCTG
5	iLOV_fwd_BamHI	GTTTCTTCGGATCCATGATTGAAAAAAACTTTGTGATTA
6	iLOV_fwd_NheI	GTTTCTTCGCTAGCATGATTGAAAAAAACTTTGTGATTA
6	iLOV_rev_HindIII	GTTTCTTCAAGCTTTTATACGTGGTCAGAACCATCCAGC
7	yfp_fwd_BamHI	GTTTCTTCGCTAGCAGTAAAGGAGAAGAACTTTTCACTG
8	yfp_rev_HindIII	GTTTCTTCAAGCTTTTATTTGTATAGTTCATCCATGCCATG
9	gfp_fwd_NheI	GTTTCTTCGCTAGCATGAGTAAAGGAGAAGAACTTTTCA
10	gfp_rev_HindIII	GTTTCTTCAAGCTTTTATTTGTACAATTCATCCATACCA
11	phage λ-tetO promoter sense strand	TCCCTATCAGTGATAGAGATTGACATCCCTATCAGTGATAGAGATACTGAGCAC
12	phage λ-tetO promoter antisense strand	GTGCTCAGTATCTCTATCACTGATAGGGATGTCAATCTCTATCACTGATAGGGA
13	Synthetic ribosome binding site	ATTAAAGAGGAGAAA
14	rrnB2 transcriptional terminator	AGAAGGCCATCCTGACGGATGGCCTTTT

Restriction sites used for cloning are underlined in the above sequences. Seven extra nucleotides are typically added upstream of the restriction sites to facilitate enymatic digestion by endonucleases.

### Protein Expression and Purification

Single colonies of *E. coli* BLR (DE3) transformants expressing the pQE80L-FbFP or pQE80L-YFP constructs were inoculated in 5 mL Lennox broth-ampicillin medium and grown for 16 hours. Cells from the overnight culture were rediluted in 500 mL medium in a 2 L shake flask. Protein expression was induced by adding isopropyl β-D-1-thiogalactopyranoside (IPTG) to a final concentration of 1 mM when the culture reached an optical density at 600 nm (A_600_) of 0.4–0.6. FbFP expression was continued for 5–6 hours at 37°C, followed by harvesting of the cells by centrifugation at 5000 g for 15 minutes at 4°C and resuspension in 10–15 mL lysis buffer (20 mM Tris hydrochloride, 200 mM sodium chloride, pH 8.0). The cells were lysed by addition of lysozyme at a concentration of 1 mg/mL and treated for 30 minutes at room temperature followed by ultrasonication (5 cycles of ten 1-second pulses of 17–20 W each). The lysate was clarified by removal of cell debris by centrifugation at 10,000 g for 20 minutes at 4°C, and the supernatant was incubated with 4 mL of nickel-nitrilotriacetic acid (Ni-NTA) resin (Qiagen) on a rocker for 1 hour at 4°C. The Ni-NTA resin and supernatant were loaded onto a gravity flow chromatographic column (Fischer Scientific) and washed with 50 mL of Ni-NTA wash buffer (20 mM Tris hydrochloride, 200 mM sodium chloride, 40 mM imidazole, pH 8.0) to remove nonspecifically bound protein. FbFPs were eluted with 20 mL Ni-NTA elution buffer (20 mM Tris hydrochloride, 200 mM sodium chloride, 500 mM imidazole, pH 8.0). FbFP fractions, which were visibly fluorescent, were pooled and loaded onto a 5 mL HiTrap Q Sepharose anion exchange column (GE Healthcare) using an automated AKTA FPLC system. The bound protein was washed with 5 column volumes (25 mL) of anion exchange wash buffer (20 mM Tris hydrochloride, 200 mM sodium chloride, pH 8.0), and eluted in 5 column volumes of anion exchange elution buffer (20 mM Tris hydrochloride, 1 M sodium chloride, pH 8.0). At NaCl concentration of 200 mM, PpFbFP and EcFbFP were strongly bound to the anion-exchange column. iLOV and YFP failed to bind to the anion exchange resin at or above 200 mM NaCl, so the salt concentrations in the Ni-NTA elution buffer and anion exchange wash buffer were reduced to 20 mM for purifying iLOV and YFP. Protein fractions were assayed for homogeneity by denaturing polyacrylamide gel electrophoresis (SDS PAGE) and fluorescence spectrometry. Protein concentrations were estimated using the Bradford assay. Purified proteins were stored at 4°C and used within a week of purification. Lysozyme was purchased from Sigma-Aldrich (St. Louis, MO). Tris hydrochloride and sodium chloride were purchased from Fisher Scientific (Pittsburgh, PA). Reagents and bovine serum albumin standards for the Bradford assay were purchased from Thermo Scientific (Rockford, IL).

### Determination of Quantum Yield

For quantum yield measurements, serial dilutions of FbFPs were prepared in anion exchange elution buffer (20 mM Tris hydrochloride, 1 M NaCl, pH 8.0), and 150 µL volumes were added to the wells of an optically clear, flat-bottom 96-well plate (Nunclon). Care was taken to ensure that the absorbance at 450 nm was less than 0.1 in order to minimize inner filtering effects. Protein samples in individual wells were excited with 450 nm light using a fluorescence spectrometer (Tecan M200), and the emission spectra were recorded between 480 and 600 nm. The emission spectrum was then integrated and normalized by the absorbance at 450 nm. Analogous measurements were made with an FMN standard, which was used as a reference with known quantum yield (QY = 0.27) to calculate FbFP quantum yields using the equation:
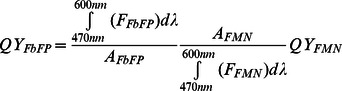
(1)where *QY* is the quantum yield, *F* is the fluorescence emission intensity, and *A* is the absorbance at 450 nm. FMN was purchased from Sigma-Aldrich (St. Louis, MO).

### Calculation of Holoprotein Fraction

FbFPs are fluorescent only in their FMN-bound (holoprotein) form, and absorbance at 450 nm is mediated by the buried FMN chromophore. Consequently, FMN-free apoprotein does not contribute to absorbance at 450 nm or fluorescence emission (**Text S1**). Therefore, the holoprotein concentration to be calculated from the Beer-Lambert equation as follows:
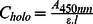
(2)where *C_holo_* is the concentration of the holoprotein, *ε* is the molar extinction coefficient of FMN (12500 M^−1^cm^−1^), and *l* is the cuvette path length. The fraction of fluorescent holoprotein in solution was determined by normalizing the holoprotein concentration by the total protein concentration estimated from the Bradford assay. In calculating the concentration of holoprotein in this way, we have verified that the concentration of free FMN in solutions of purified FbFPs to be negligible (**Text S1**).

### Determination of Oligomeric State

Oligomeric states of FbFPs were determined by gel filtration chromatography using a Superdex 200 column in an AKTA FPLC system (GE Healthcare). The column was calibrated with globular proteins standards, including bovine thyroglobulin (670 kDa), bovine γ-globulin (158 kDa), chicken ovalbumin (44 kDa), and horse myoglobin (17 kDa). Purified FbFPs were loaded in the column and washed with 50 mL phosphate buffered saline or anion exchange elution buffer (20 mM Tris hydrochloride, 1M NaCl, pH 8.0) (**Figure S1**). Elution volumes corresponding to peaks in the 280 nm absorption chromatogram were recorded and molecular mass was estimated from a calibration graph of the logarithm of molecular mass of standards plotted against corresponding peak elution volumes. Molecular mass of a monomer was calculated assuming an average mass of 110 Da per amino acid. Oligomeric state was determined by dividing the estimated molecular mass by the calculated mass of a monomer. Molecular weight standards were purchased from Biorad (Hercules, CA) and phosphate buffered saline was purchased from Fisher Scientific (Pittsburgh, PA).

### Effect of pH on FbFP Fluorescence

Buffers of varying pH were prepared using 200 mM disodium hydrogen phosphate/100 mM sodium citrate (pH 2–8) and 100 mM sodium carbonate/100 mM sodium bicarbonate solutions (pH 9–11) at the following concentrations: pH 2 (4 mM Na_2_HPO_4_,/98 mM sodium citrate), pH 4 (77.1 mM Na_2_HPO_4_,/61.45 mM sodium citrate), pH 6 (126.3 mM Na_2_HPO_4_/36.85 mM sodium citrate), pH 8 (194.5 mM Na_2_HPO_4_, 2.75 mM sodium citrate), pH 10 (60 mM Na_2_CO_3_, 40 mM NaHCO_3_), and pH 11 (90 mM Na_2_CO_3_, 10 mM NaHCO_3_). Purified FbFP preparations in anion exchange elution buffer (pH 8.0) were diluted 1∶30 in 450 µL of the respective pH-adjusted buffers and 200 µL sample volumes were added to each well of a flat-bottom 96-well plate (Nunclon). We verified that the buffer pH remained constant at the desired value subsequent to titration of FbFP using a pH meter (Accumet, ColeParmer). FbFPs were incubated for 2.5 hours in the respective buffers and emission spectra were recorded between 480 and 600 nm every 30 minutes using a fluorescence spectrometer (Tecan M200) with gain setting manually set at 50. Excitation and emission slit widths were set to 9 nm and 20 nm respectively. In the case of YFP, excitation wavelength was set at 505 nm and emission was recorded at 528 nm. At each pH, fluorescence intensities corresponding to the emission wavelength (495 nm) were normalized by the maximum emission intensity recorded at physiological pH. All chemicals were purchased from Sigma-Aldrich (St. Louis, MO).

### Effect of Temperature on FbFP Fluorescence

In order to assess the thermal stability of FbFPs, 300 µL volumes of purified protein preparations were added to 6×50 mm borosilicate glass culture tubes (Kimble Chase, Vineland, NJ) housed in custom-made aluminum holders. The glass tube/holder assembly was then placed in a fluorescence spectrometer (Cary Varian), which was heated to the desired temperature using a temperature controlled water bath (Cary Varian). Evaporative loss was minimized by sealing the mouth of the glass tubes with heat resistant tape or adding 10 µL BacLight mounting oil (Invitrogen) to the surface of the protein solution in the cuvette. FbFPs were incubated at different temperatures (40°C, 50°C, 60°C, and 70°C) for up to 2.5 hours, and fluorescence emission spectra were recorded between 470 and 600 nm every 30 minutes. Excitation and emission slit widths were set at 5 nm and the photomultiplier tube gain was set to medium (600 V). In the case of YFP, excitation was provided at 500 nm and emission spectra were scanned between 515 and 600 nm. For each temperature, the fluorescence intensity corresponding to the emission wavelength (495 nm) was normalized to the emission intensity at 495 nm measured at room temperature.

### Fluorescence Recovery after Denaturation

300 µL volumes of purified FbFPs were added to 6×50 mm borosilicate glass culture tubes (Kimble Chase, Vineland, NJ) housed in custom-made aluminum holders. The glass tube/aluminum assemblies were then placed in a fluorescence spectrometer (Cary Varian), which was heated to 90°C or 70°C. FbFPs were denatured by heating at 90°C or 70°C for 25 minutes. Denaturation was monitored by the loss of peak fluorescence emission at 495 nm and the appearance of a 525 nm emission peak characteristic for free FMN. Following denaturation, protein samples were cooled to 25°C, and renaturation was monitored by recording the emission spectra between 470 and 600 nm. Excitation and emission slit widths were set at 5 nm and the photomultiplier tube gain was set to medium (600 V).

### Effect of Strong Reducing Agents on FbFP Fluorescence

In order to probe the sensitivity of FbFPs to reducing conditions, 200 µL of protein samples were added to the wells of a 96-well plate and treated with a strong reductant (sodium dithionite) at 25 mM concentration. Protein solutions were excited with 450 nm light in a fluorescence spectrometer (Tecan M200), and emission spectra were recorded between 480 and 600 nm every 30 minutes for a period of 2.5 hours with gain setting manually set at 50. Excitation and emission slit widths were set to 9 nm and 20 nm respectively. Fluorescence intensities corresponding to the emission wavelength (495 nm) were normalized by the fluorescence intensity at 495 nm of purified proteins incubated without sodium dithionite.

### Monitoring Promoter Activity in E. coli Using FbFPs as Reporters

FbFPs were evaluated as potential reporters for real-time measurements of transcriptional activity from IPTG-inducible phage T5-lacO [49] and constitutive phage lambda-tetO [52] hybrid promoters in *E. coli* MG1655 cells. Single colonies of *E. coli* cells expressing the FbFP of interest were inoculated in M9 minimal medium supplemented with 20 mM glucose or 0.5% glycerol as the carbon source and grown for 16 hours with vigorous shaking (200 r.p.m.) at 37°C. Following overnight growth, cells were inoculated at 1% dilution in 250 µL of the relevant medium (M9-glucose, or M9-glycerol) in an optically clear, flat-bottom, 96-well plate (Nunclon). The growth medium was supplemented with IPTG to express the T5-lacO promoter. We employed an IPTG concentration that yielded the highest levels of normalized fluorescence for the respective reporters - specifically, 1 mM (for iLOV), 0.1 mM (for PpFbFP and EcFbFP) and 10 mM (for venus). In the case of the phage lambda transcriptional constructs, IPTG was eliminated because the promoter is constitutive in wild type *E. coli*. The 96-well plate was incubated with shaking (3.5 mm amplitude, linear shaking) in a fluorescence spectrometer (Tecan M200) at 37°C. Media evaporation was minimized through the use of covered 96-well plates. *E. coli* cells in each well were periodically illuminated with 450 nm light and transcription from the promoter was followed by measuring fluorescence intensity at the emission wavelength (495 nm) every 15–20 minutes over a period of 16 hours. Cell growth was simultaneously monitored by measuring absorbance at 600 nm (*A_600nm_*). Excitation and emission slit widths were set at 9 nm and 20 nm respectively and gain was manually adjusted to 50. Fluorescence measurements from the induced cultures were normalized by the corresponding *A_600nm_* values and normalized background fluorescence measured in uninduced cells was subtracted. In an analogous fashion, promoter activities were also determined using YFP or GFP. *E. coli* cells expressing YFP were excited at 505 nm and emission intensity was recorded at 530 nm. *E. coli* cells expressing GFP were excited at 485 nm and emission intensity was recorded at 510 nm. Promoter activity profiles elucidated using FbFPs as reporters were compared with transcriptional profiles derived using YFP or GFP. We restricted our analysis of gene expression to the exponential growth phase of *E. coli* cells, which corresponds to the optical density range from 0.4 to 0.8, determined by absorbance at 600 nm. Exponential phase growth rates were estimated from the slope of a linear regression between log[optical density at 600 nm] versus time (*µ*). Doubling times were calculated using the equation:
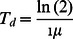
(3)


## Supporting Information

Figure S1 **Size exclusion chromatograms of FbFPs.**
(DOCX)

Figure S2 **Emission spectra of FbFPs at pH 6–7.**
(DOC)

Figure S3 **Denaturation of PpFbFP and EcFbFP at pH 2.**
(DOC)

Figure S4 **Effect of pH and temperature on fluorescence in YFP.**
(DOC)

Figure S5 **Denaturation of PpFbFP and EcFbFP at 70°C.**
(DOC)

Figure S6 **Denaturation/renaturation of EcFbFP and YFP monitored using fluorescence emission.**
(DOC)

Figure S7 **Effect of strong reducing conditions on FbFP fluorescence.**
(DOC)

Figure S8 **Doubling times of** ***E. coli*** **expressing FbFPs from inducible and constitutive promoters.**
(DOC)

Figure S9 **Complete transcriptional profiles of P_T5-lacO_ promoter in** ***E. coli*** **cells grown in M9-glucose.**
(DOC)

Figure S10 **Complete transcriptional profiles of P_T5-lacO_ promoter in** ***E. coli*** **cells grown in M9-glycerol.**
(DOC)

Figure S11 **Intracellular expression of PpFbFP, EcFbFP, and YFP assessed using western blotting.**
(DOC)

Figure S12 **Transcriptional profiles of P_T5-lacO_ promoter in** ***E. coli*** **assessed using LVA tagged FbFPs.**
(DOC)

Figure S13 **Complete Transcriptional profiles of P_L-tetO_ promoter in** ***E. coli.***
(DOC)

Text S1 **Calculation of holoprotein fraction in solutions of purified FbFPs.**
(DOCX)

Text S2 **Comparison of FMN-binding pockets between EcFbFP and iLOV.**
(DOCX)

Text S3 **Relative quantum yields of FbFPs as a function of pH, temperature, redox, and in anaerobic conditions.**
(DOCX)

Text S4 **Nucleotide sequences of FbFP genes.**
(DOCX)
