# Supplementary material for: Characterization of Flavin-Based Fluorescent Proteins: An Emerging Class of Fluorescent Reporters
Source: PLoS One. 2013 May 31;8(5):e64753. doi: 10.1371/journal.pone.0064753 (PMC3669411; doi:10.1371/journal.pone.0064753)
Supplement: Figure S1 — Size exclusion chromatograms of FbFPs. (DOCX) [file pone.0064753.s001.docx]

**Size exclusion chromatograms of FbFPs**

***V_elution_* = 15.5 mL**

Absorbance at 280 nm (A.U.)

0.8

0.6

0.4

0.2

**A**

Elution volume (mL)

**B**

***V_elution_* = 15.6 mL**

Absorbance at 280 nm (A.U.)

0.6

0.4

0.2

0.8

Elution volume (mL)

**C**

***V_elution_*  = 17.4 mL**

Absorbance at 280 nm (A.U.)

0.6

0.4

0.2

0.8

Elution volume (mL)

**Figure S1**. FbFPs were loaded on a size-exclusion column using PBS (0.8 % NaCl) as the running buffer. *V_elution_* corresponding to *A_280 nm_* were recorded for A) PpFbFP, B) EcFbFP, and C) iLOV
