# Supplementary material for: Characterization of Flavin-Based Fluorescent Proteins: An Emerging Class of Fluorescent Reporters
Source: PLoS One. 2013 May 31;8(5):e64753. doi: 10.1371/journal.pone.0064753 (PMC3669411; doi:10.1371/journal.pone.0064753)
Supplement: Figure S2 — Emission spectra of FbFPs at pH 6–7. (DOC) [file pone.0064753.s002.doc]

**Emission spectra of FbFPs at pH 6 and 7**

**A
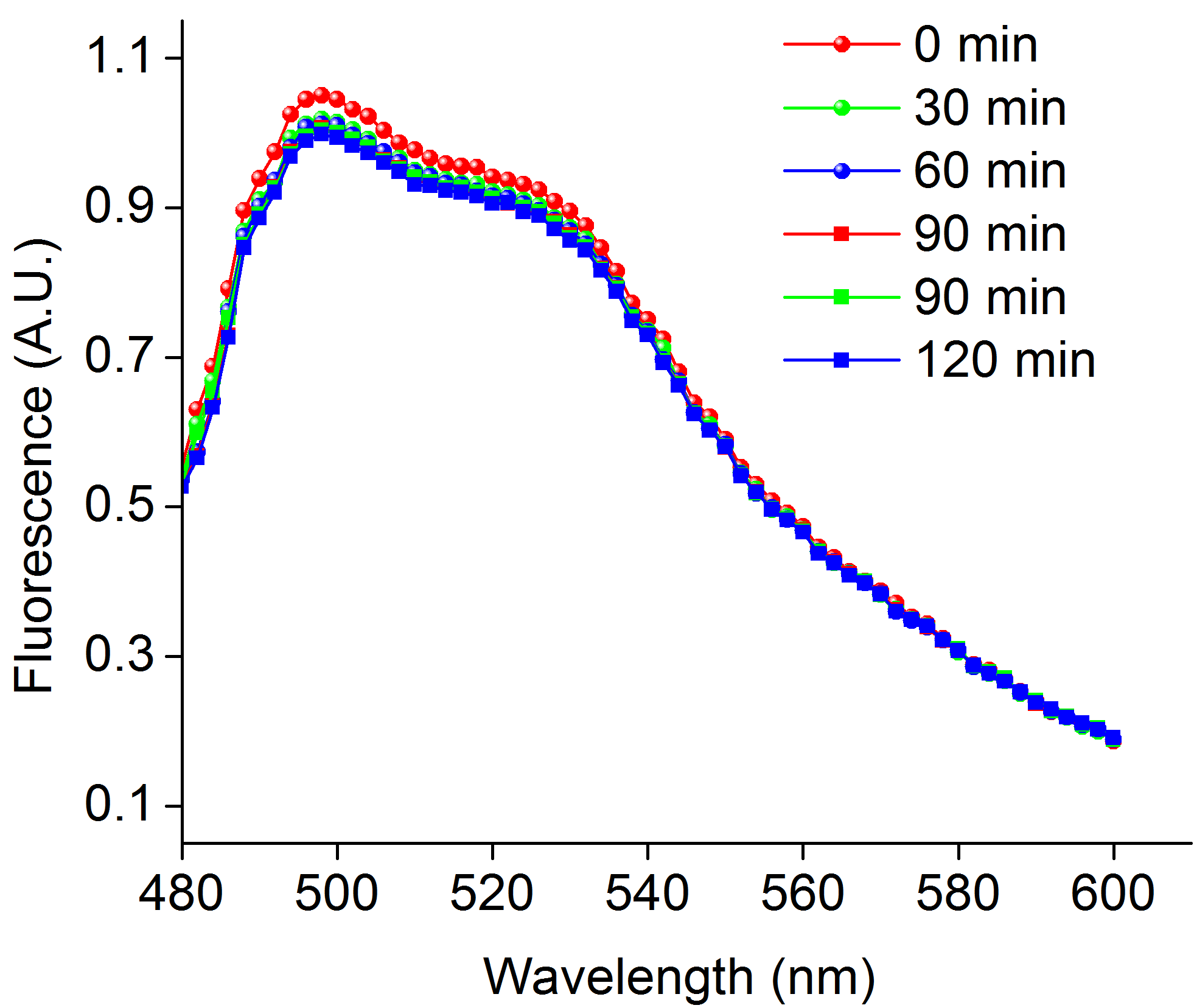
**

**C
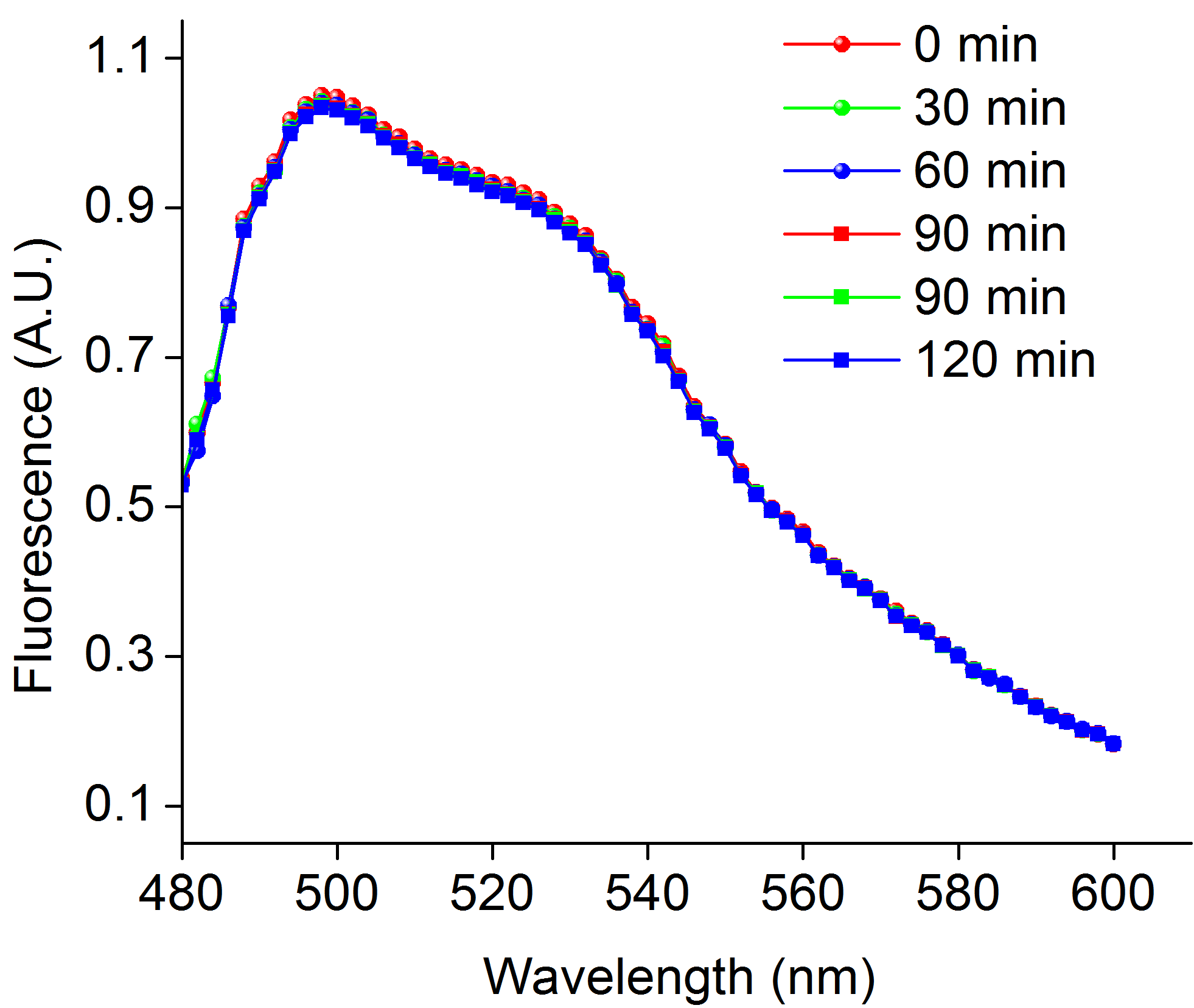
**

**Figure S2**. Fluorescence emission from FbFPs is maximum at pH 6-7 and is relatively stable at these pH values. A) PpFbFP, B) EcFbFP, and C) iLOV were incubated at pH 7 (PpFbFP and EcFbFP) or pH 6 (iLOV) for 2 h. and emission spectra were recorded following excitation at 450 nm.

**B
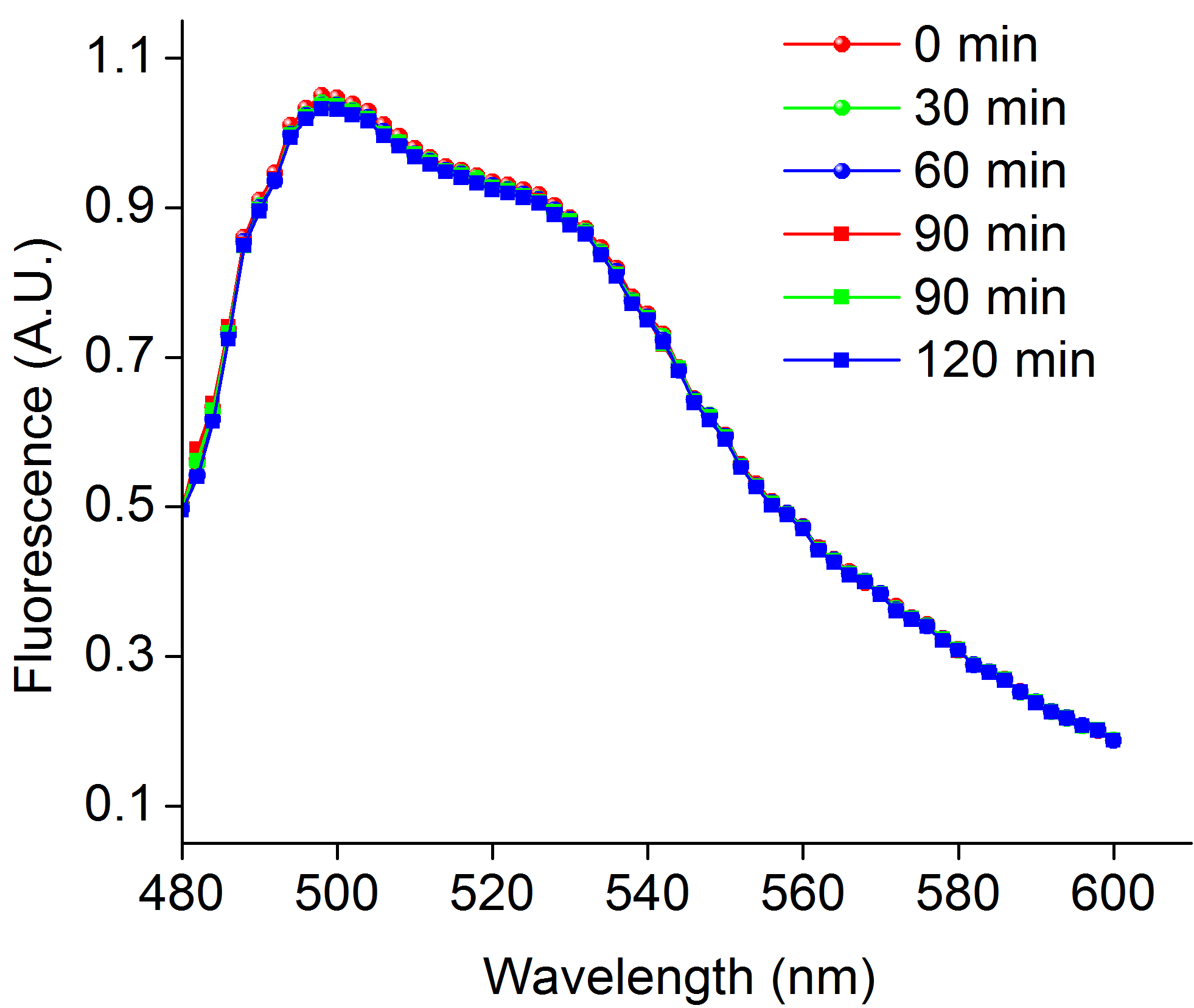
**
