# Supplementary material for: Characterization of Flavin-Based Fluorescent Proteins: An Emerging Class of Fluorescent Reporters
Source: PLoS One. 2013 May 31;8(5):e64753. doi: 10.1371/journal.pone.0064753 (PMC3669411; doi:10.1371/journal.pone.0064753)
Supplement: Figure S3 — Denaturation of PpFbFP and EcFbFP at pH 2. (DOC) [file pone.0064753.s003.doc]

**Denaturation of PpFbFP and EcFbFP at pH 2**

**A**

**
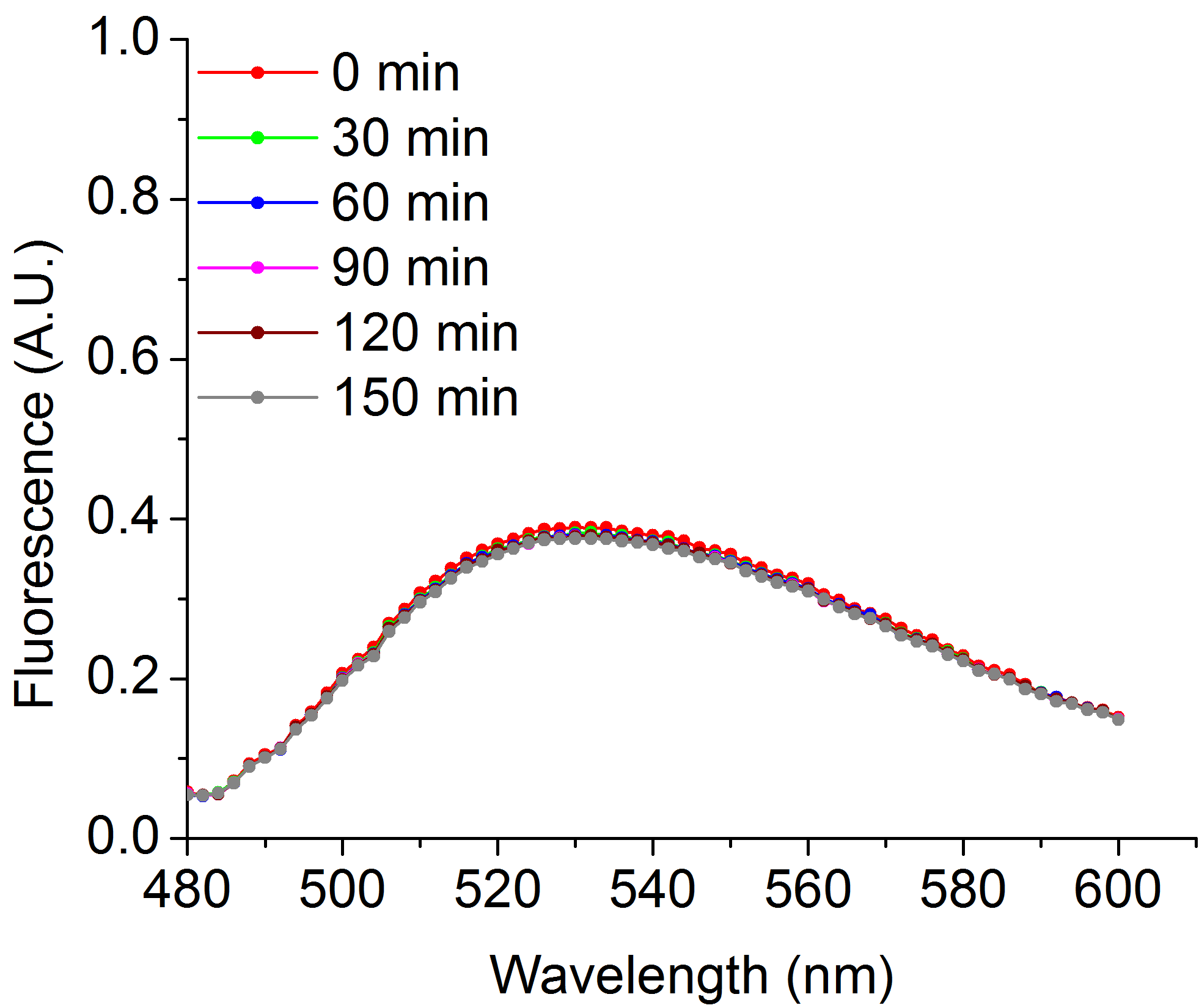
**

**Figure S3**. FbFPs are readily denatured at pH 2. A) PpFbFP and B) EcFbFP were incubated at pH 2 for 2.5 h. and emission spectra were recorded following excitation at 450 nm. Denaturation was marked by a loss of the FbFP emission spectrum and the appearance of an FMN-emission spectrum with a peak at 525 nm.

**B**

**
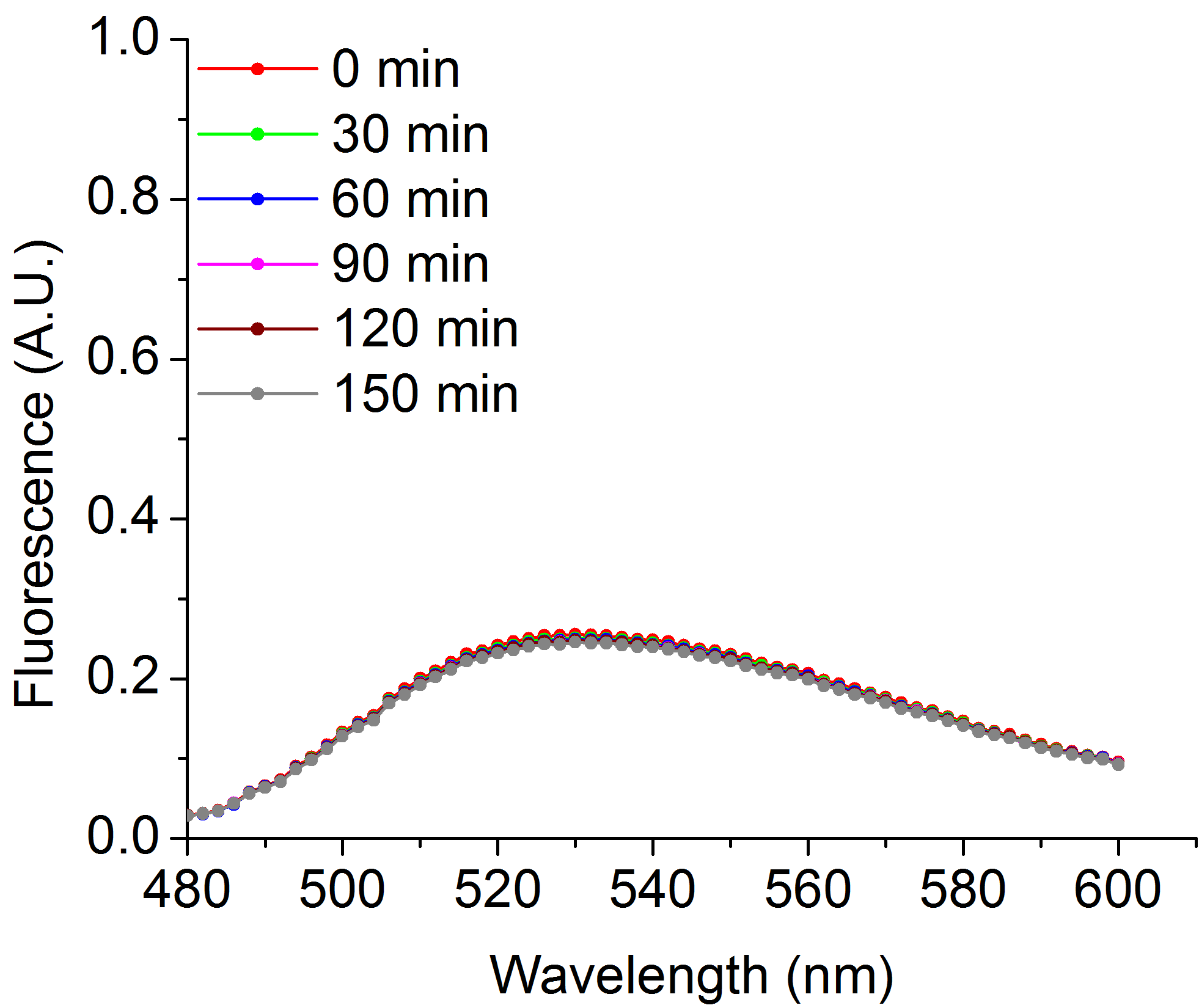
**
