# Supplementary material for: Characterization of Flavin-Based Fluorescent Proteins: An Emerging Class of Fluorescent Reporters
Source: PLoS One. 2013 May 31;8(5):e64753. doi: 10.1371/journal.pone.0064753 (PMC3669411; doi:10.1371/journal.pone.0064753)
Supplement: Figure S4 — Effect of pH and temperature on fluorescence in YFP. (DOC) [file pone.0064753.s004.doc]

**Effect of pH and temperature on fluorescence in YFP**

**A**

**
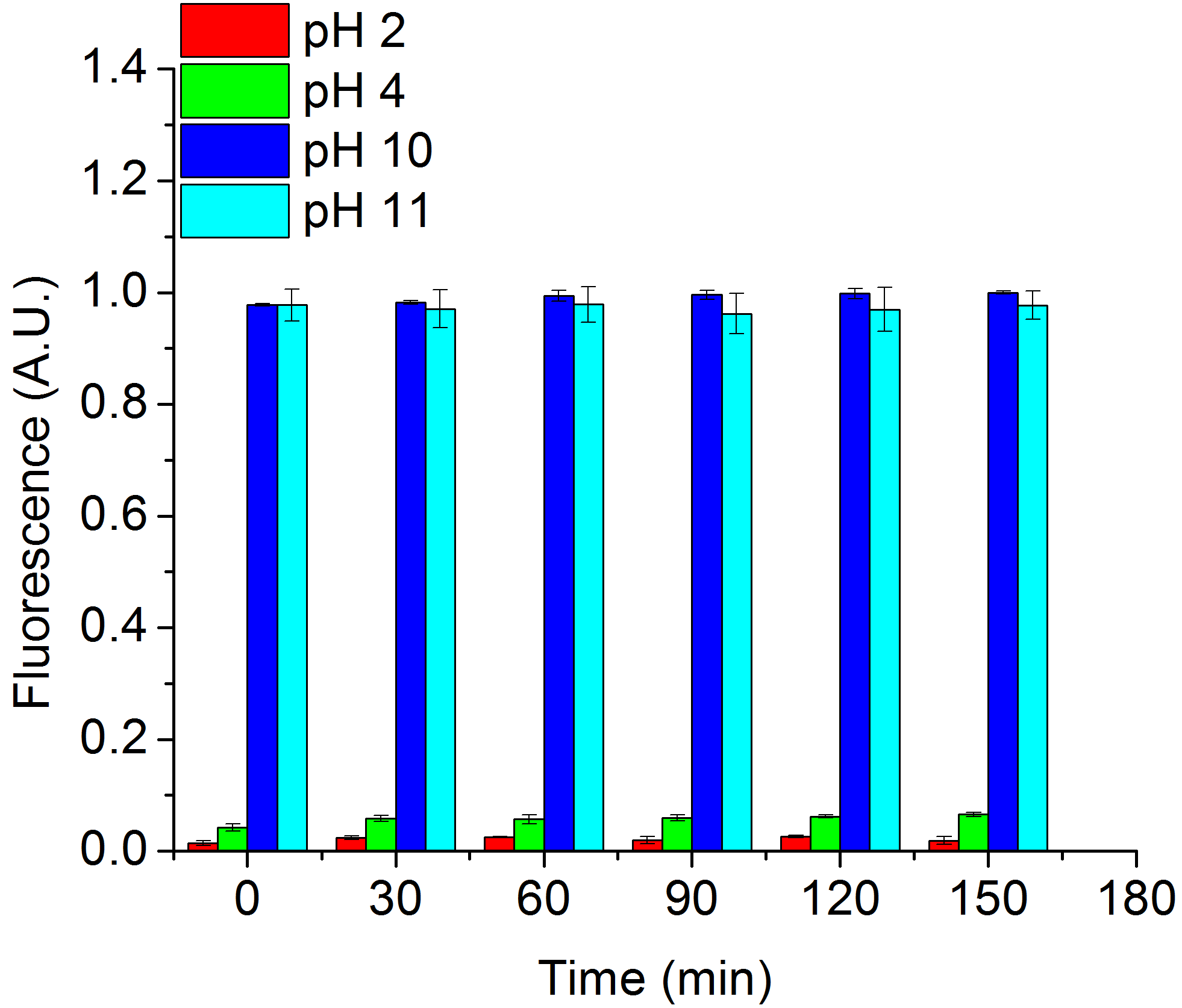
**

**B**

**
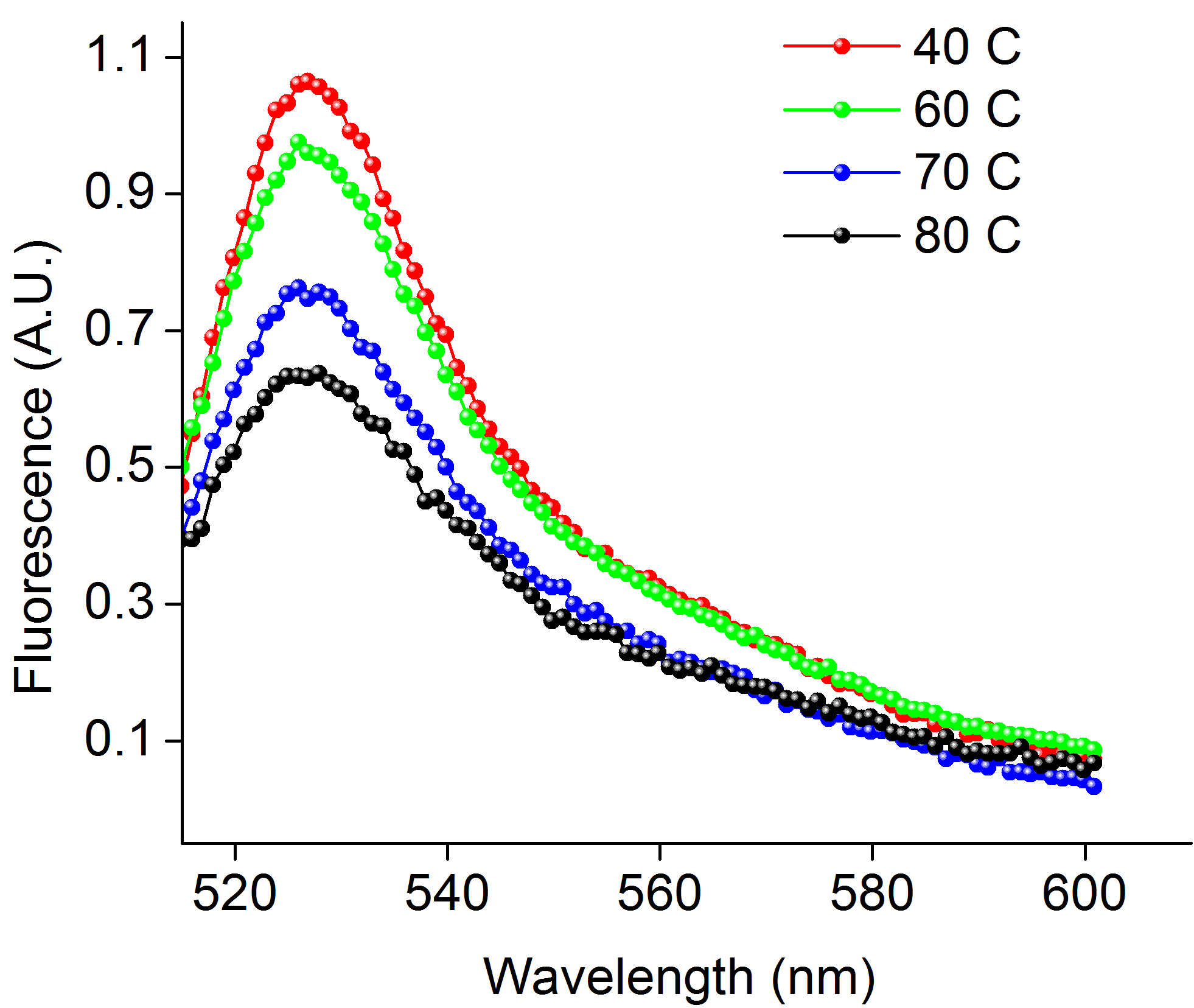
**

**Figure S4**. A) Histograms depict the fraction of peak fluorescence (measured at 528 nm at physiological pH corresponding to peak emission) retained by YFP after incubation in buffers of pH 2, 4, 10, and 11 for 2.5 h. B) YFP is characterized by excellent thermostability retaining upto 50 % of its maximum fluorescence (measured at room temperature) upon prolonged incubation for 2.5 h. at 80 ºC.
