# Supplementary material for: Characterization of Flavin-Based Fluorescent Proteins: An Emerging Class of Fluorescent Reporters
Source: PLoS One. 2013 May 31;8(5):e64753. doi: 10.1371/journal.pone.0064753 (PMC3669411; doi:10.1371/journal.pone.0064753)
Supplement: Figure S6 — Denaturation/renaturation of EcFbFP and YFP monitored using fluorescence emission. (DOC) [file pone.0064753.s006.doc]

**Denaturation/renaturation of EcFbFP and YFP monitored using fluorescence emission**

**A B**

**
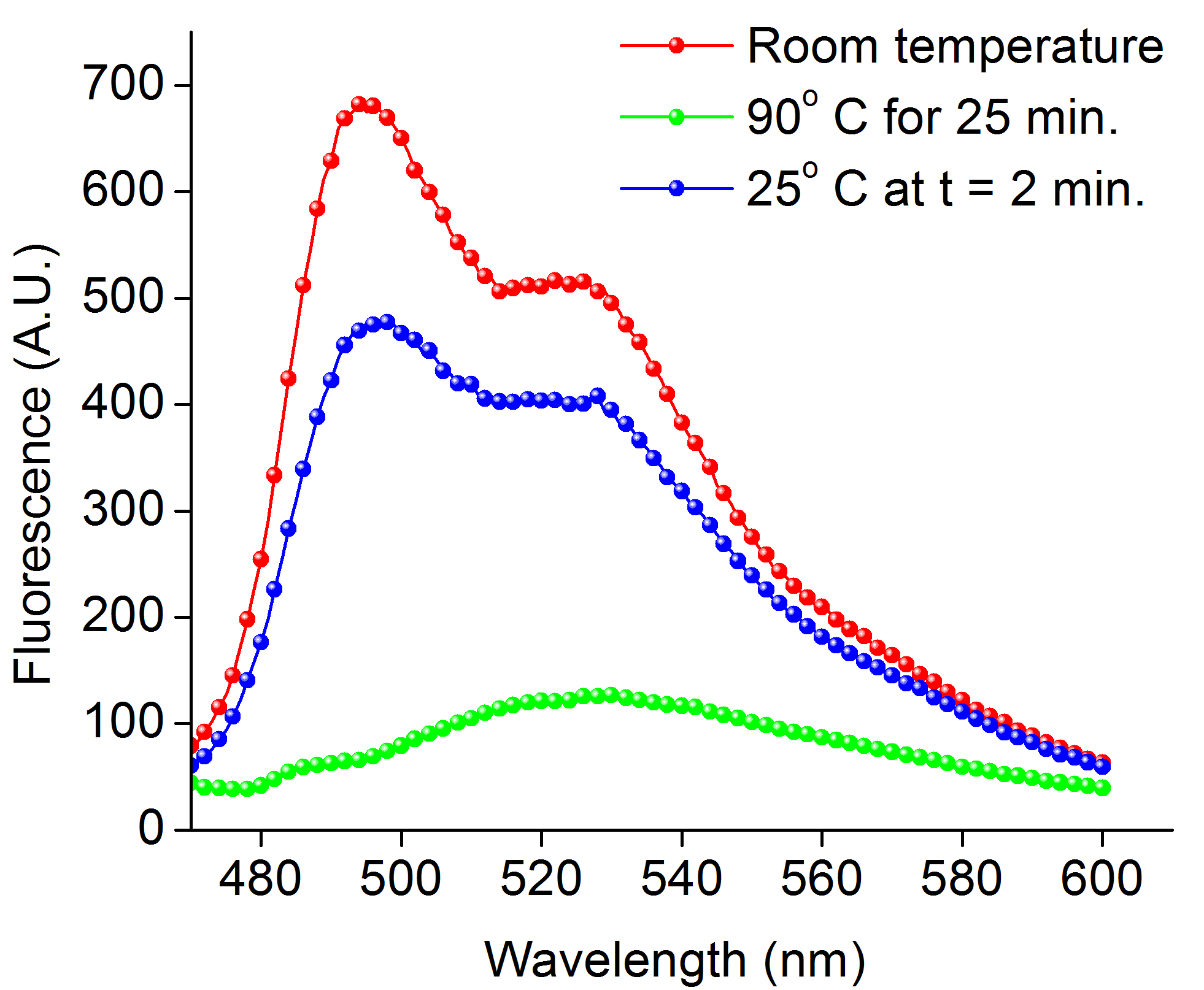
**

**
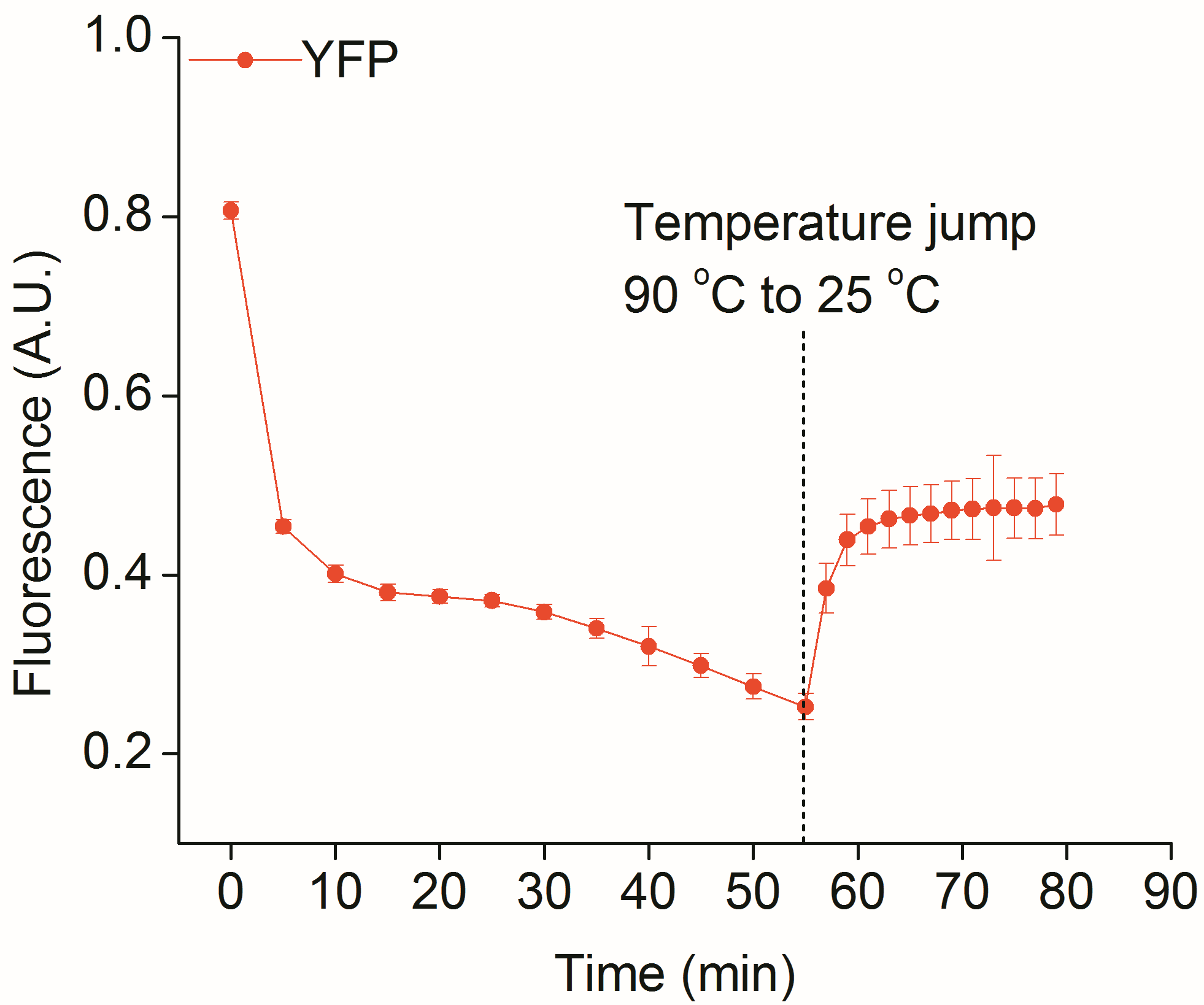
**

**Figure S6**. (A) EcFbFP and (B) YFP were denatured by heating at 90 ºC for 25 minutes and 55 minutes respectively and unfolding was monitored based on progressive loss in peak fluorescence emission at 495 nm (EcFbFP) or 530 nm (YFP). In case of EcFbFP, the characteristic FbFP spectrum (shown in red) was replaced by an emission spectrum typical of free FMN (shown in green). Upon rapid cooling to room temperature (in 2-3 minutes), EcFbFP was readily renatured and the FbFP-specific emission spectrum (shown in blue) was restored. Renaturation in YFP was incomplete and only 46 % of YFP fluorescence was restored in the same time that it took for EcFbFP to regain greater than 90 % of its initial fluorescence signal.
