# Supplementary material for: Characterization of Flavin-Based Fluorescent Proteins: An Emerging Class of Fluorescent Reporters
Source: PLoS One. 2013 May 31;8(5):e64753. doi: 10.1371/journal.pone.0064753 (PMC3669411; doi:10.1371/journal.pone.0064753)

**Effect of strong reducing conditions on FbFP fluorescence**

**Figure S7**. Histograms depict the fraction of peak fluorescence (measured at 495 nm in 20 mM Tris, 1 M NaCl, pH 8.0 buffer) retained by FbFPs after incubation with 25 mM sodium dithionite (reductant) for 2.5 h.


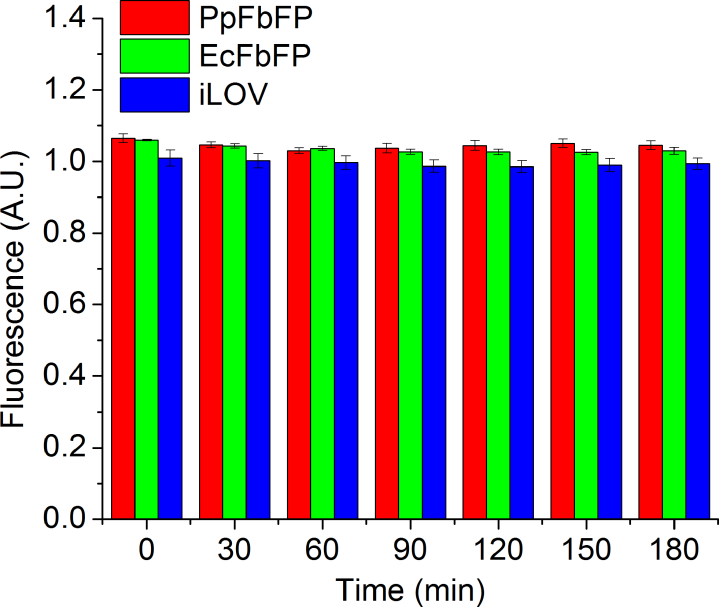

Supplement: Figure S7 — Effect of strong reducing conditions on FbFP fluorescence. (DOC) [file pone.0064753.s007.doc]
