# Supplementary material for: Characterization of Flavin-Based Fluorescent Proteins: An Emerging Class of Fluorescent Reporters
Source: PLoS One. 2013 May 31;8(5):e64753. doi: 10.1371/journal.pone.0064753 (PMC3669411; doi:10.1371/journal.pone.0064753)
Supplement: Figure S8 — Doubling times of E. coli expressing FbFPs from inducible and constitutive promoters. (DOC) [file pone.0064753.s008.doc]

**Doubling times of *E. coli* expressing FbFPs from inducible and constitutive promoters**

**A
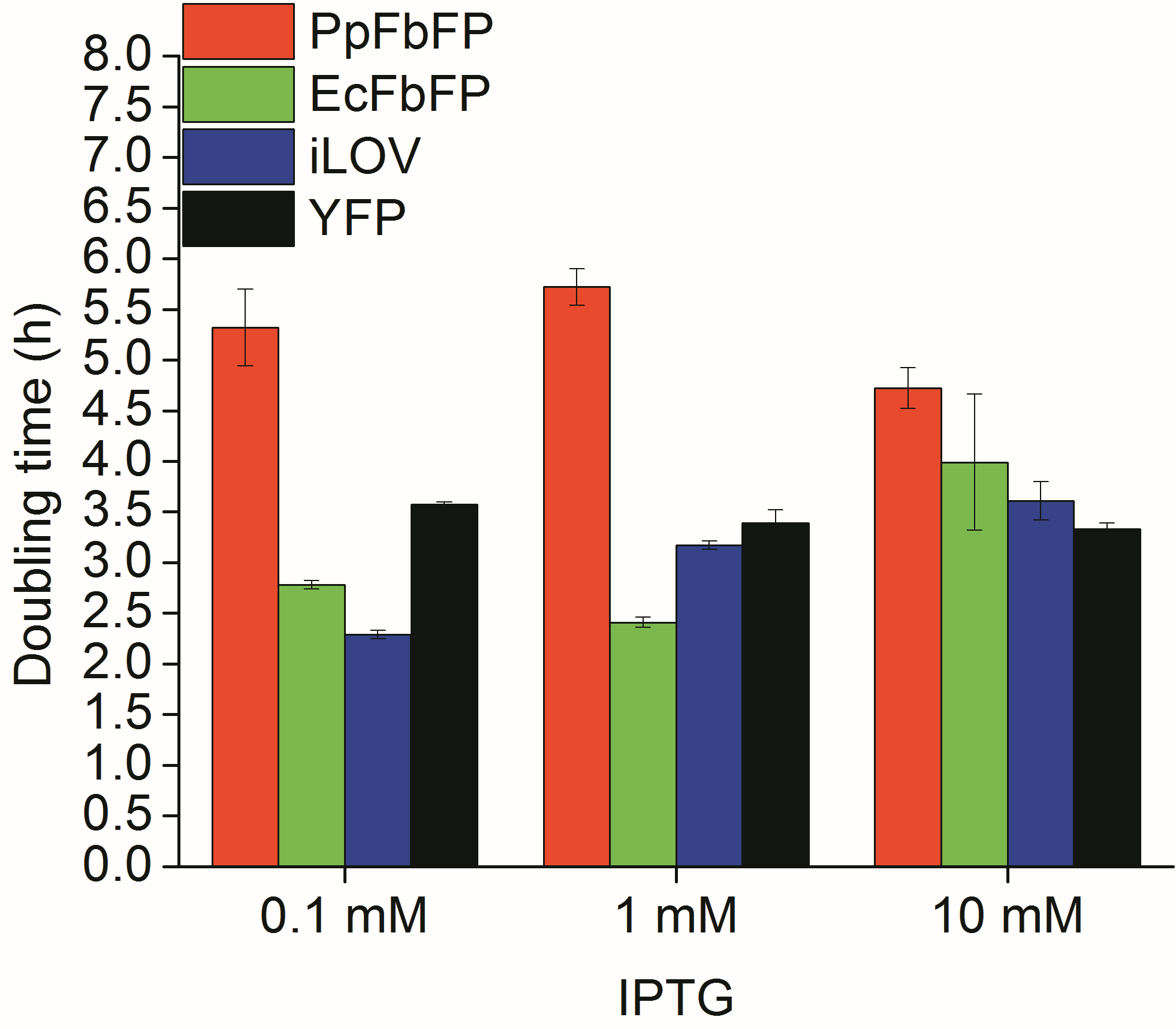
**

**B**

**
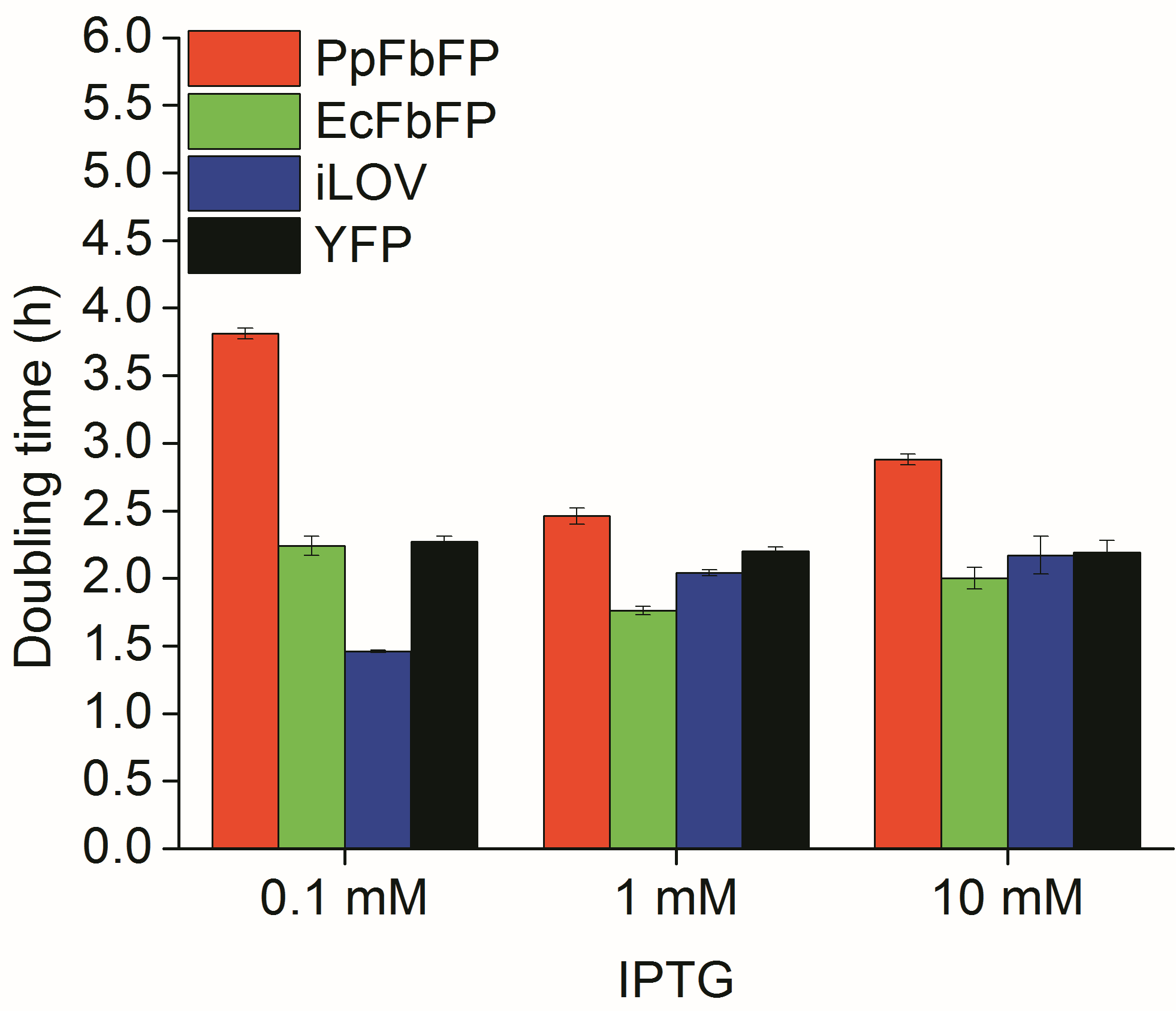
**

**C
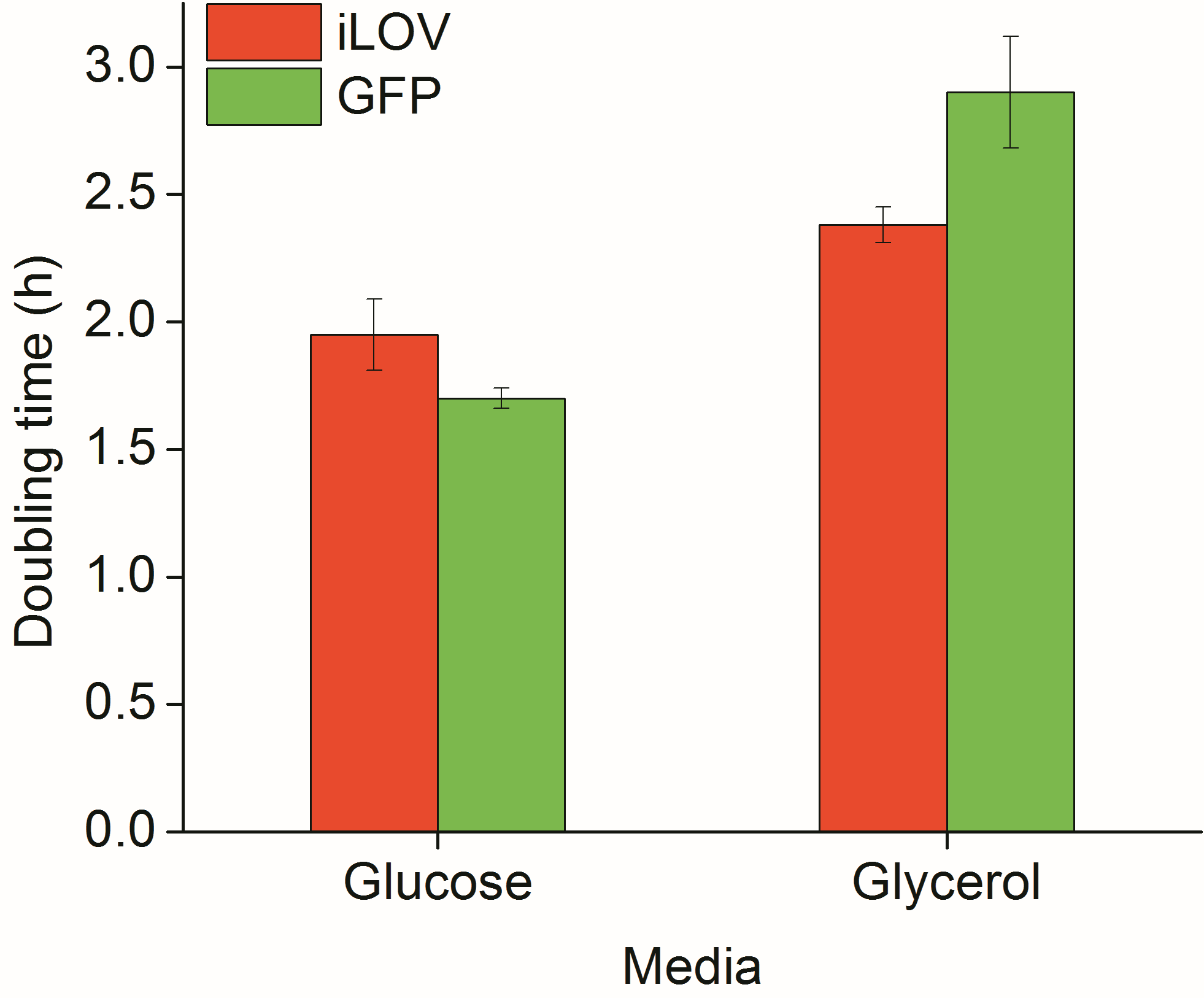
**

**Figure S8**. FbFPs (and YFP) were transcriptionally fused to an IPTG-inducible phage T5-lacO hybrid promoter and expressed in *E. coli* MG1655 cells. Protein expression was induced using IPTG at varying concentrations, which was added to a 1 % inoculum of an overnight culture in M9 medium using A) glucose and B) glycerol as the carbon. Doubling times were calculated over the logarithmic phase of cell growth corresponding to 0.4 < *A_600 nm_* < 0.8. C) Transcriptional fusions were constructed between iLOV (and GFP) and a phage lambda P_L_-tetO hybrid promoter that is constitutive in wild type *E. coli*. Overnight cultures were diluted at 1 % in fresh M9 medium supplemented with glucose or glycerol as the carbon source and doubling times were calculated as before.
