# Supplementary material for: Characterization of Flavin-Based Fluorescent Proteins: An Emerging Class of Fluorescent Reporters
Source: PLoS One. 2013 May 31;8(5):e64753. doi: 10.1371/journal.pone.0064753 (PMC3669411; doi:10.1371/journal.pone.0064753)

**Complete transcriptional profiles of P_T5-lacO_ promoter in *E. coli* cells grown in M9-glucose**

**A**


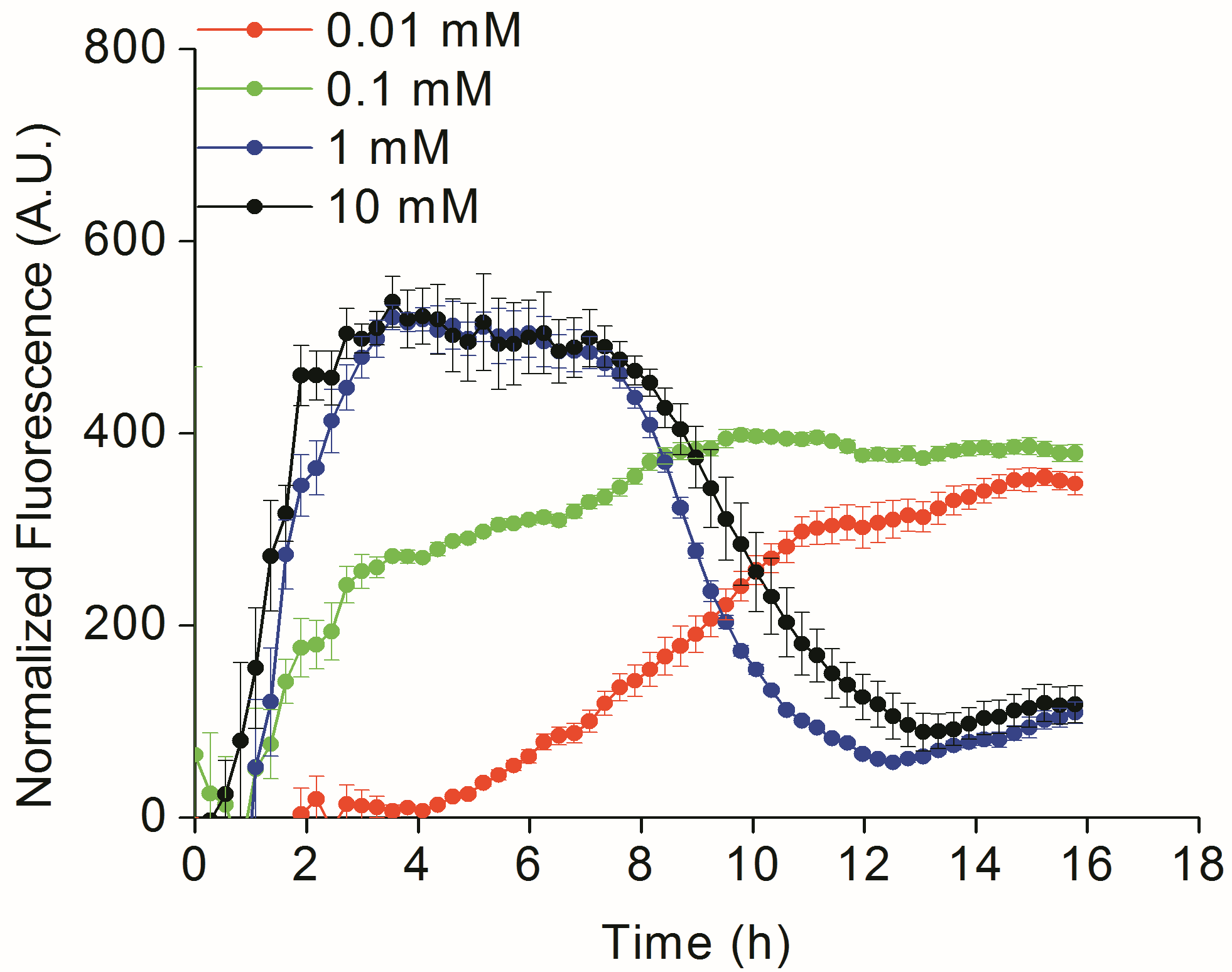


**C**


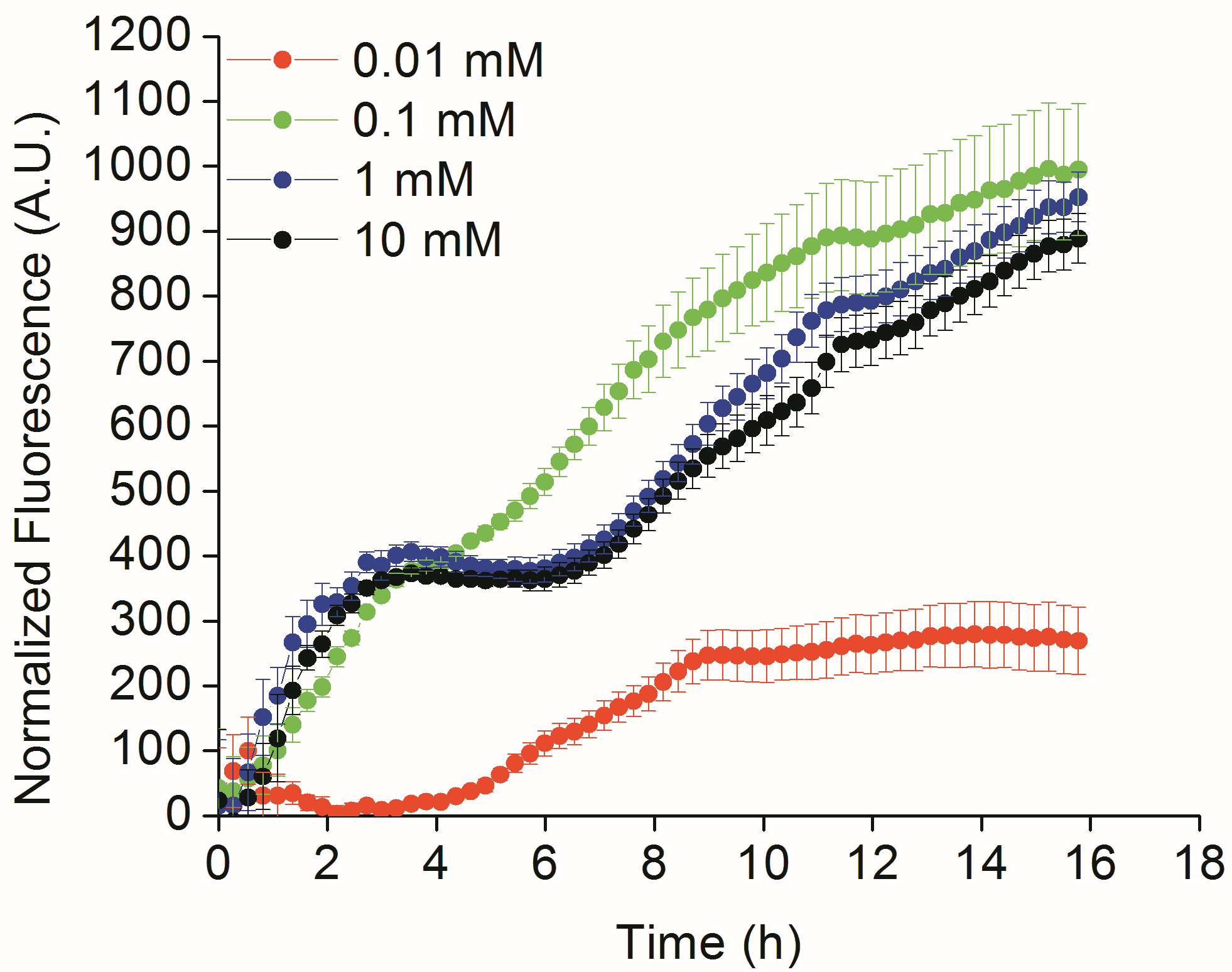


**Figure S9**. *E. coli* MG1655 cells expressing (A) PpFbFP, (B) EcFbFP, (C) iLOV, or (D) YFP under transcriptional control of an IPTG-inducible bacteriophage T5-lacO hybrid promoter (P_T5-lacO_) were cultured in M9 minimal media using glucose as the carbon source. Promoter activity was induced using different concentrations of IPTG – 0.01 mM, 0.1 mM, 1 mM, and 10 mM. Promoter activities were monitored by periodically measuring fluorescence emission at 495 nm upon excitation at 450 nm (FbFPs) or at 530 nm upon excitation at 505 nm (YFP). Fluorescence measurements were normalized by the optical density at 600 nm (*A_600 nm_*).

**B**


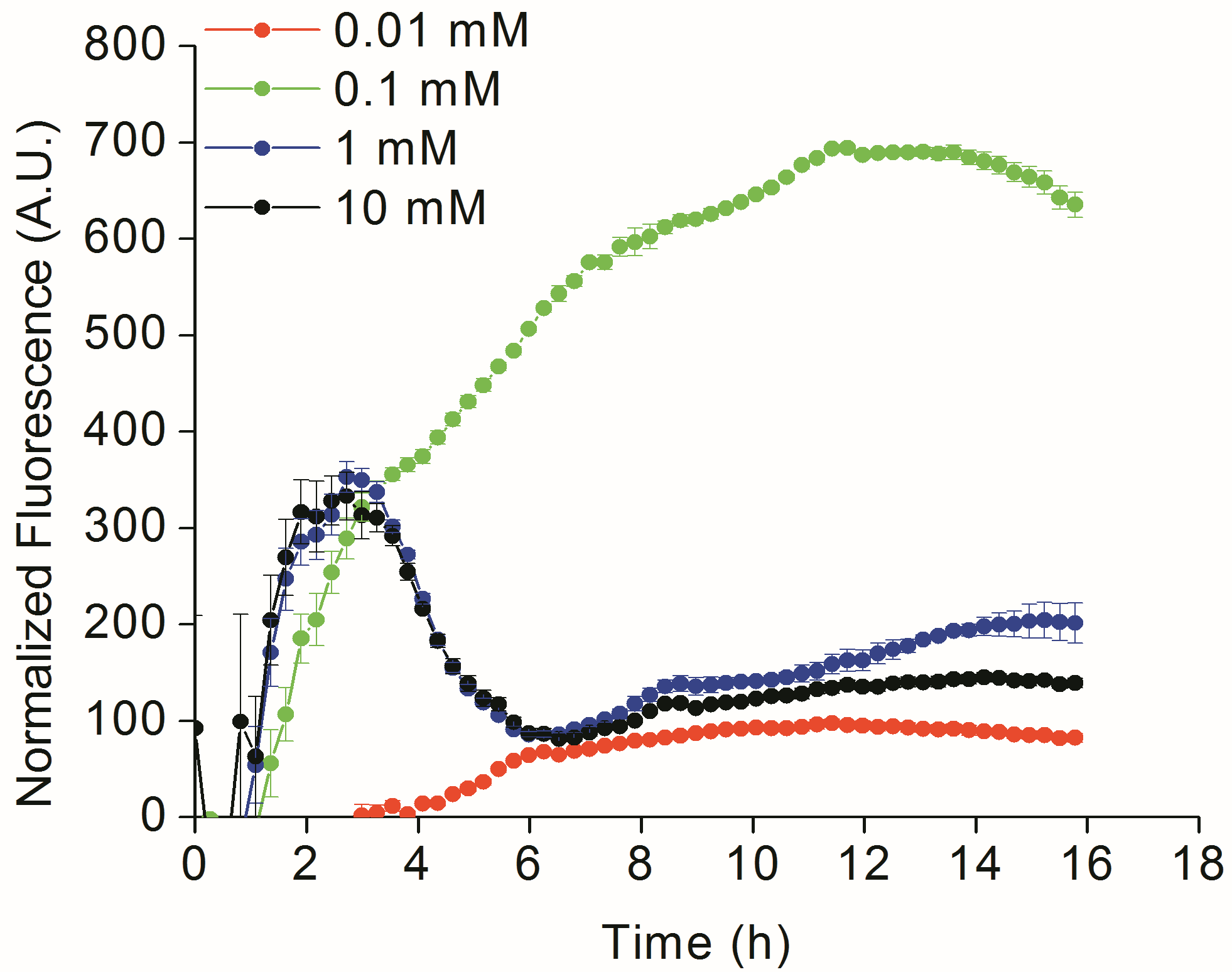


**D**


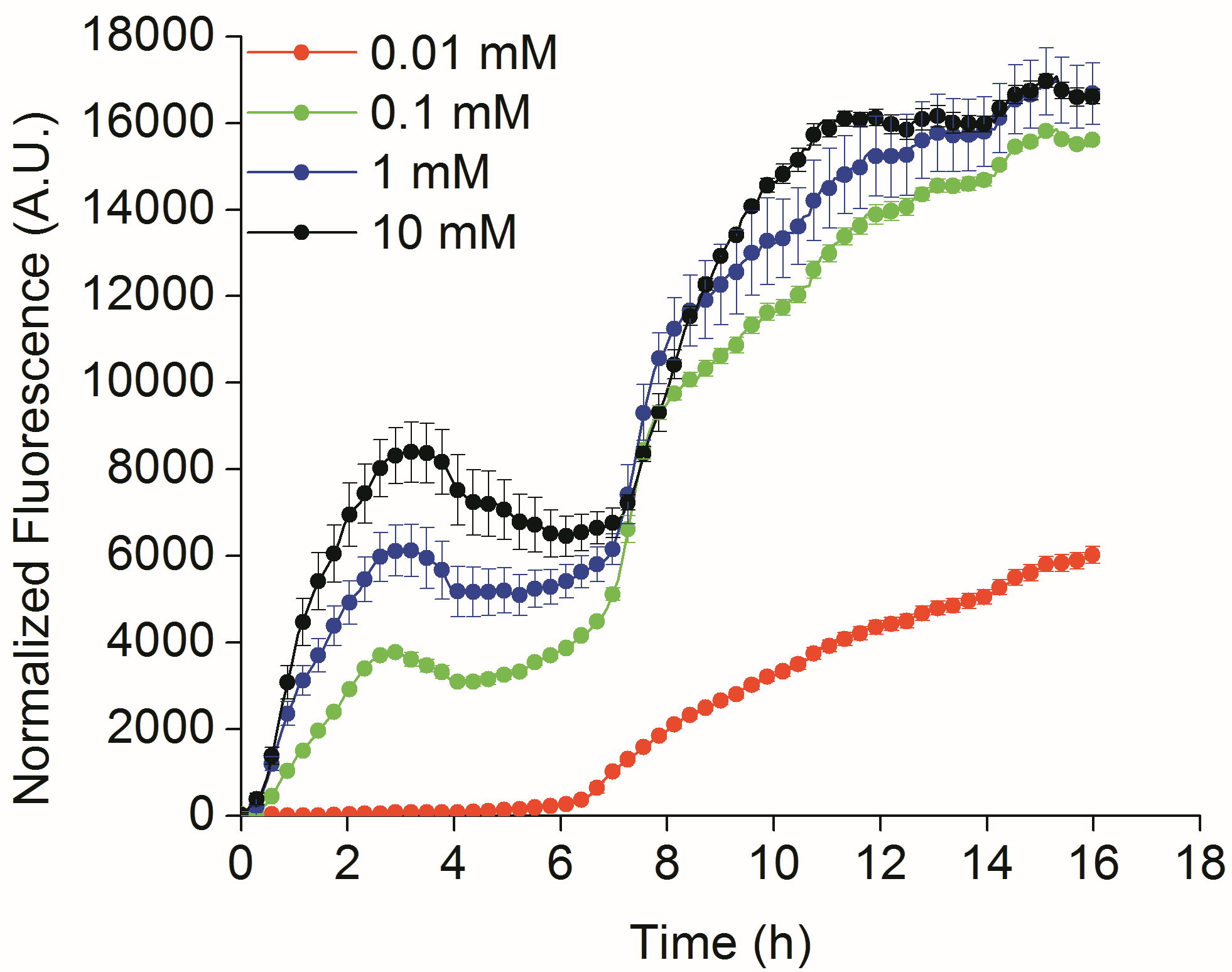

Supplement: Figure S9 — Complete transcriptional profiles of PT5-lacO promoter in E. coli cells grown in M9-glucose. (DOC) [file pone.0064753.s009.doc]
