# Supplementary material for: Characterization of Flavin-Based Fluorescent Proteins: An Emerging Class of Fluorescent Reporters
Source: PLoS One. 2013 May 31;8(5):e64753. doi: 10.1371/journal.pone.0064753 (PMC3669411; doi:10.1371/journal.pone.0064753)
Supplement: Figure S11 — Intracellular expression of PpFbFP, EcFbFP, and YFP assessed using western blotting. (DOC) [file pone.0064753.s011.doc]

**Intracellular expression of PpFbFP, EcFbFP, and YFP assessed using western blotting**


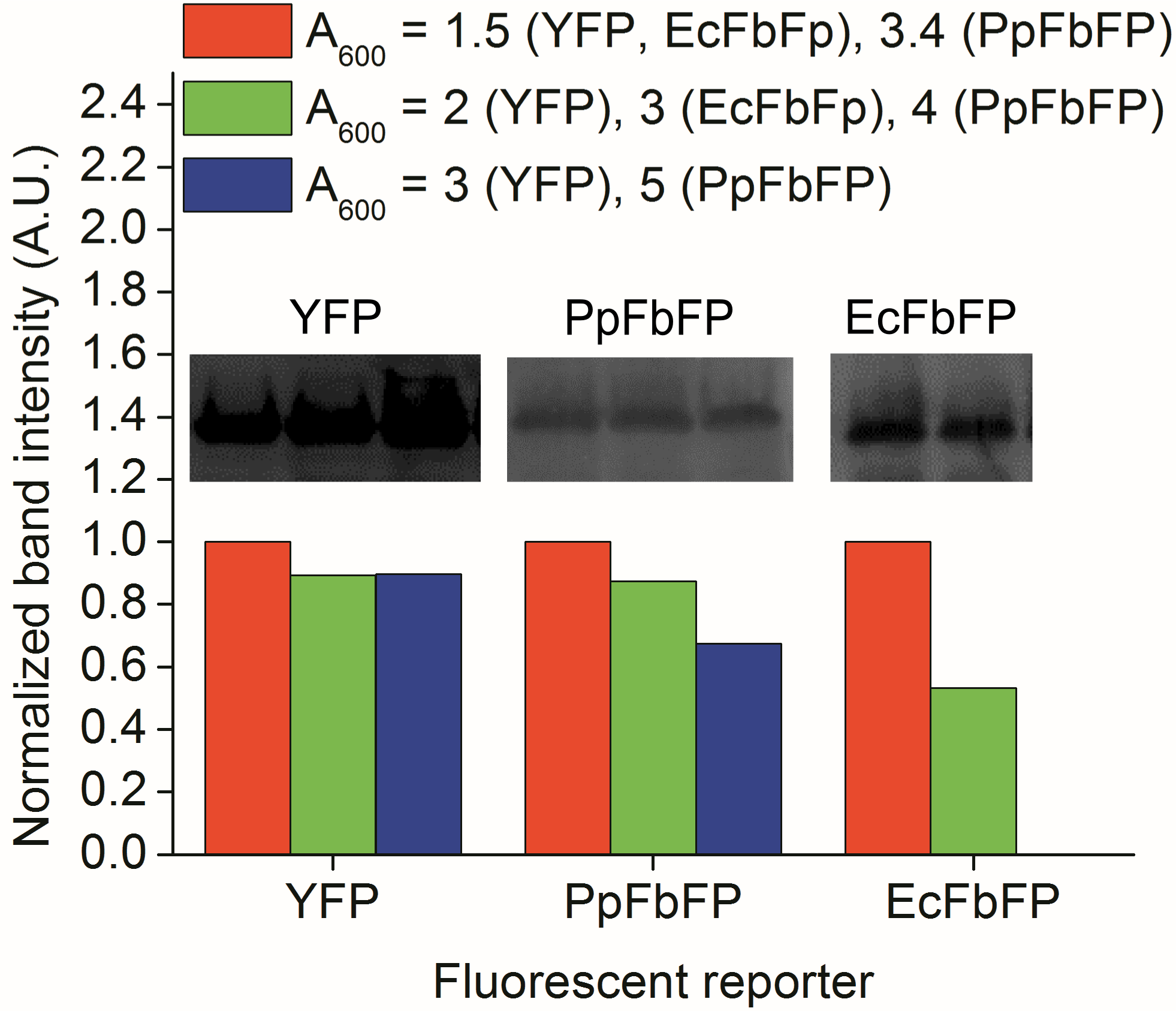


**Figure S11***. E. coli* MG1655 cells respectively transformed with PpFbFP, EcFbFP, and YFP were induced for expression of the respective proteins using IPTG at 1 mM concentration in M9-glucose medium. Cell samples were collected at 2-3 time points corresponding to the exponential phase of growth. Protein expression was probed by western blotting using rabbit anti-PpFbFP, rabbit anti-EcFbFP, or mouse anti-His_6_ primary antibodies against His_6_-tagged YFP. Expression was quantified by integrating intensity of bands for YFP, PpFbFP, and EcFbFP. Proteins were detected using horse radish peroxidase conjugated goat anti-rabbit and anti-mouse secondary antibodies and a chemifluorescent acridinium substrate (ECL Plus Western Blotting, GE). We observed that YFP expression was nearly invariant that is consistent with fluorescence measurements and confirms steady state dynamics of the T5 promoter. However, protein degradation was observed in the case of PpFbFP and EcFbFP.
