# Supplementary material for: Characterization of Flavin-Based Fluorescent Proteins: An Emerging Class of Fluorescent Reporters
Source: PLoS One. 2013 May 31;8(5):e64753. doi: 10.1371/journal.pone.0064753 (PMC3669411; doi:10.1371/journal.pone.0064753)
Supplement: Figure S12 — Transcriptional profiles of PT5-lacO promoter in E. coli assessed using LVA tagged FbFPs. (DOC) [file pone.0064753.s012.doc]

**Transcriptional profiles of P_T5-lacO_ promoter in *E. coli* assessed using LVA tagged FbFPs**

**
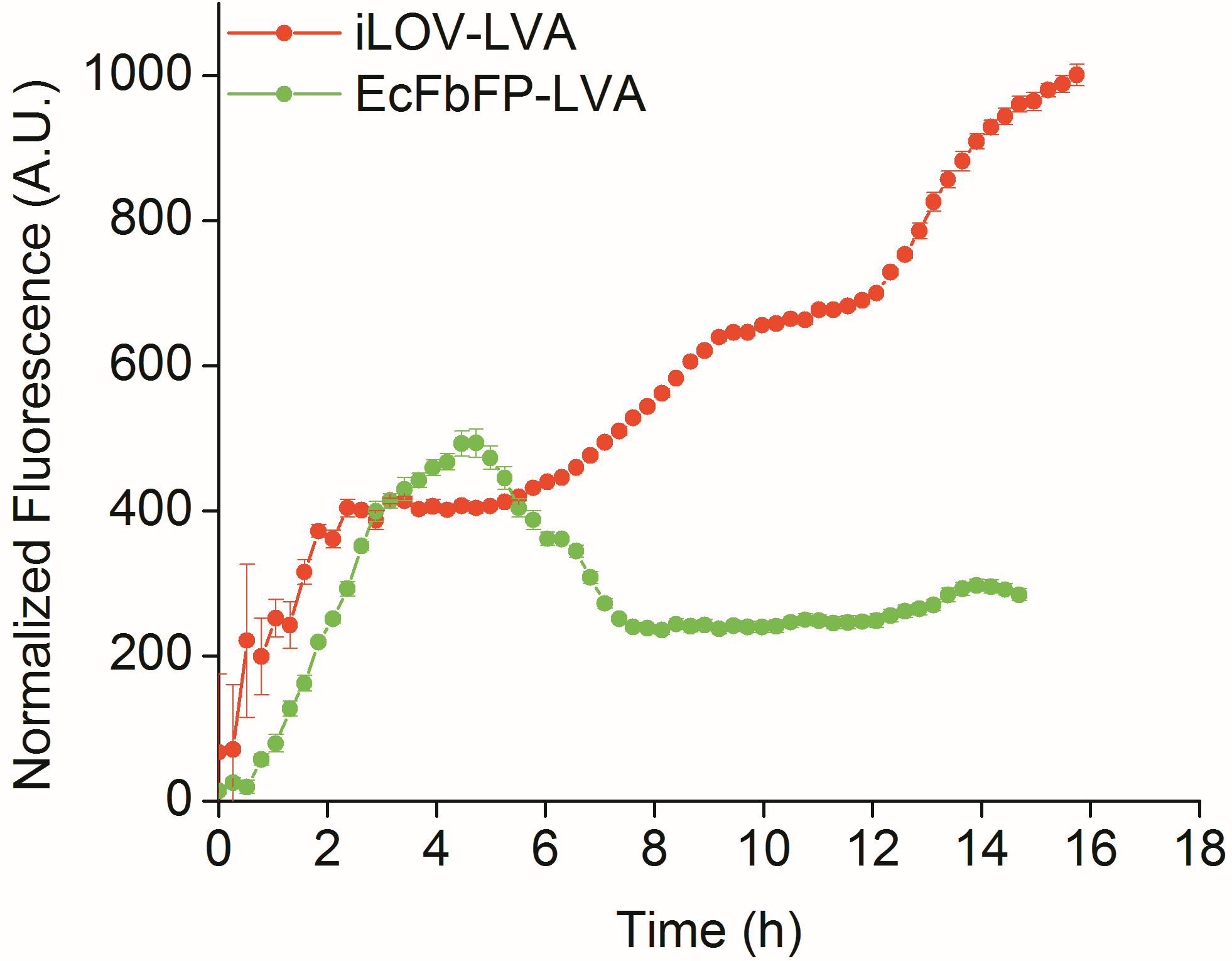
**

**Figure S12**. *E. coli* MG1655 cells expressing LVA-tagged protease sensitive iLOV or EcFbFP under transcriptional control of an IPTG-inducible bacteriophage T5 P_T5-lacO_ hybrid promoter were cultured in M9 minimal media using glucose or glycerol as the carbon source. Expression was induced using 0.5 mM IPTG. Promoter activities were monitored by periodically measuring fluorescence emission at 495 nm upon excitation at 450 nm (FbFPs). Fluorescence measurements were normalized by the optical density at 600 nm (*A_600 nm_*).
