# Supplementary material for: Characterization of Flavin-Based Fluorescent Proteins: An Emerging Class of Fluorescent Reporters
Source: PLoS One. 2013 May 31;8(5):e64753. doi: 10.1371/journal.pone.0064753 (PMC3669411; doi:10.1371/journal.pone.0064753)
Supplement: Figure S13 — Complete Transcriptional profiles of PL-tetO promoter in E. coli. (DOC) [file pone.0064753.s013.doc]

**Complete Transcriptional profiles of P_L-tetO_ promoter in *E. coli***

**
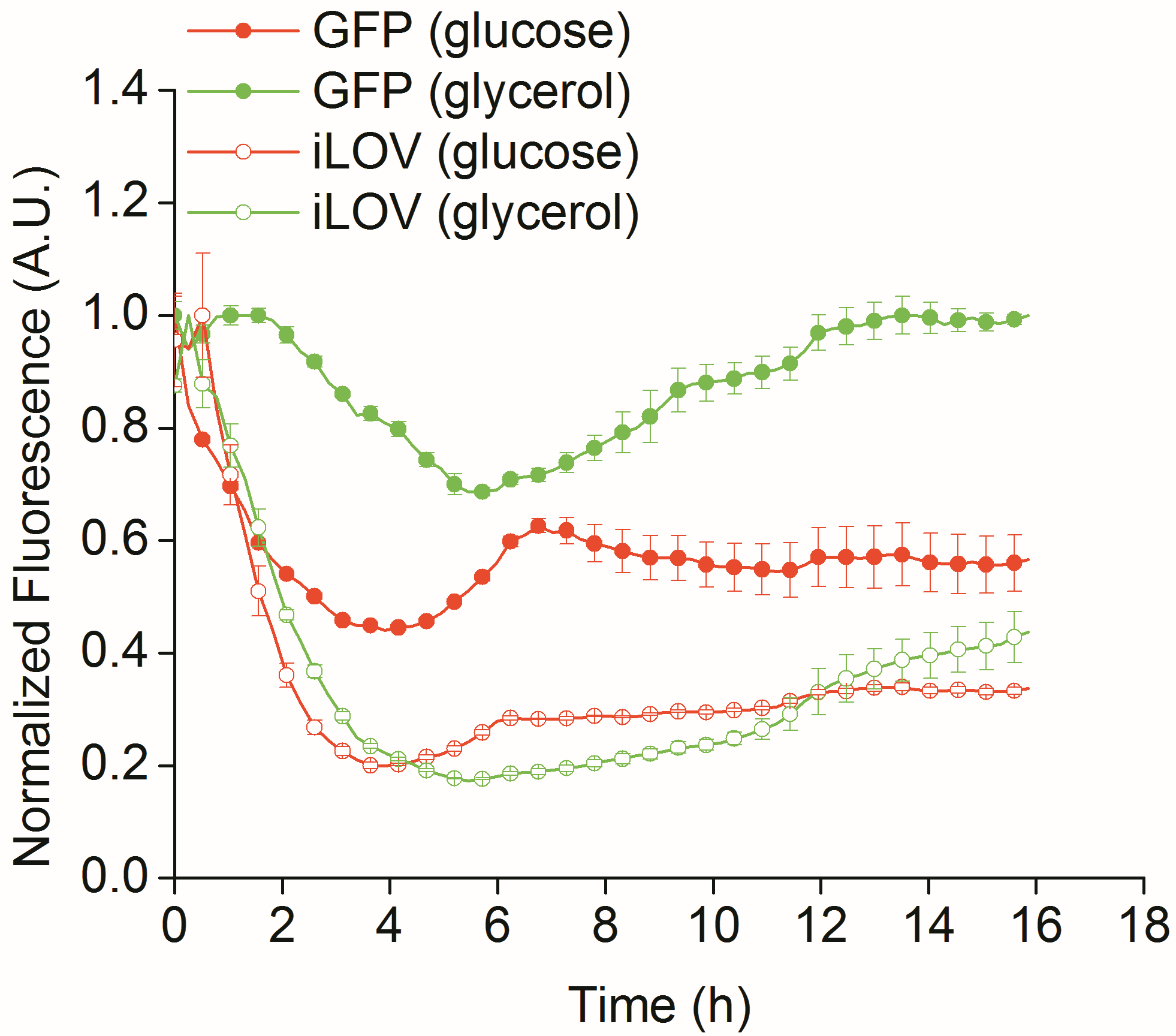
**

**Figure S13**. *E. coli* MG1655 cells expressing iLOV or GFP under transcriptional control of a constitutive bacteriophage lambda P_L-tetO_ hybrid promoter were cultured in M9 minimal media using glucose or glycerol as the carbon source. Promoter activities were monitored by periodically measuring fluorescence emission at 495 nm upon excitation at 450 nm (FbFPs) or at 530 nm upon excitation at 505 nm (YFP). Fluorescence measurements were normalized by the optical density at 600 nm (*A_600 nm_*).
