# Supplementary material for: Characterization of Flavin-Based Fluorescent Proteins: An Emerging Class of Fluorescent Reporters
Source: PLoS One. 2013 May 31;8(5):e64753. doi: 10.1371/journal.pone.0064753 (PMC3669411; doi:10.1371/journal.pone.0064753)
Supplement: Text S1 — Calculation of holoprotein fraction in solutions of purified FbFPs. (DOCX) [file pone.0064753.s014.docx]

**Calculation of holoprotein fraction in solution of purified FbFPs**

Firstly, we note that the T5 promoter used to express the FbFPs is a very strong promoter; using this promoter to express FbFPs, the intracellular levels of FbFP are likely to substantially exceed cellular FMN pools, which may contribute to a higher fraction of FMN-free apo-FbFP in purified protein preparations. In general, the holoprotein fraction in solutions of purified FbFPs is likely affected by the protein expression and purification conditions. In this work, expression, purification, and assays for holoprotein fraction were conducted under identical conditions for iLOV, EcFbFP, and PpFbFP. Therefore, we anticipate that the relative levels of holoprotein between the different FbFPs to remain similar. We calculate holoprotein fraction in solutions of purified FbFPs based on the absorption of holo-FbFP at 450 nm. Our calculation relies on the following assumptions: 1) apo-FbFP does not absorb substantially at 450 nm and 2) concentration of free FMN in a solution of FbFP is negligible, which makes holo-FbFP the sole species with an absorbance peak at 450 nm.

**Purification of apo-FbFP**

We verified the first assumption by purifying apo-FbFP and demonstrating minimal absorbance at 450 nm. Apo formulations of FbFPs were prepared by urea denaturation of FbFPs immobilized on a nickel chelating column. Specifically, FbFPs were expressed in *E. coli* BLR cells as described and loaded on a nickel chelating column (Qiagen). The column was washed with 50 mL of Ni-NTA wash buffer (20 mM Tris hydrochloride, 200 mM sodium chloride, 40 mM imidazole, pH 8.0). FbFPs bound to the nickel chelating resin were then incubated with denaturation buffer (20 mM Tris hydrochloride, 200 mM sodium chloride, 8 M urea, 2.5 mM β-mercapto ethanol, pH 8.0) at room temperature for 24 - 48 h. Subsequently, the denaturation buffer was drained off and the apo-proteins were eluted using Ni-NTA elution buffer (20 mM Tris hydrochloride, 200 mM sodium chloride, 500 mM imidazole, pH 8.0). Apo-FbFP was concentrated using centrifugal spin columns with a 10 kDa molecular weight cutoff (Amicon Ultra-4, Millipore).

**Concentration of free FMN in purified FbFP solutions**

In order to estimate the concentration of free FMN in a solution of purified iLOV, we first deflavinated a solution of iLOV and compared the concentration of total FMN (free FMN + FMN released by deflavination) to the total protein concentration. Deflavination of iLOV was achieved using trichloroacetic acid precipitation. Specifically, purified iLOV was supplemented with trichloroacetic acid at 10% v/v and incubated on ice for 20-30 min. Precipitated proteins were pelleted by centrifugation at 13,000 r.p.m. for 15 min. The supernatant was collected and assayed for FMN using absorbance measurements at 450 nm. Flavin concentrations were determined using a calibration curve of absorbance at 450 nm versus concentration of flavin. In case of a high concentration of free flavin in solution, the flavin concentration would be expected to substantially exceed the total protein concentration. However, flavin concentrations (≈ 20 µM) were consistently lower than iLOV concentrations (≈ 24 µM). Based on these results, we estimate the fraction of fluorescent holoprotein in solution using the Beer-Lambert law as described in the main text (**Materials and Methods**).
