# Supplementary material for: Characterization of Flavin-Based Fluorescent Proteins: An Emerging Class of Fluorescent Reporters
Source: PLoS One. 2013 May 31;8(5):e64753. doi: 10.1371/journal.pone.0064753 (PMC3669411; doi:10.1371/journal.pone.0064753)
Supplement: Text S2 — Comparison of FMN-binding pockets between EcFbFP and iLOV. (DOCX) [file pone.0064753.s015.docx]

**Comparison of FMN-binding pockets between EcFbFP and iLOV**

FMN-binding pockets in EcFbFP and iLOV were defined to include amino acids that are directly involved in hydrophobic interactions or hydrogen bonding with the isoalloxazine ring of the FMN-chromophore. The amino acids along with their temperature factor values (B-factor) are listed in the following table.

**Table S1. B-factor values of amino acids in FMN-binding pocket in iLOV and EcFbFP**

| **EcFbFP**  **(PDB ID: 2PR5)** | **B-factor** | **iLOV**  **(PDB ID: 4EES)** | **B-factor** | **Nature of interaction with FMN** |
| --- | --- | --- | --- | --- |
| V28 | 20.38 | V7 | 17.68 | hydrophobic |
| T30 | 16.39 | T9 | 17.33 | hydrophobic |
| L65 | 21.54 | L44 | 25.09 | hydrophobic |
| V75 | 14.83 | I57 | 24.35 | hydrophobic |
| I78 | 16.08 | I61 | 20.62 | hydrophobic |
| R79 | 14.18 | L85 | 21.63 | hydrophobic |
| L82 | 17.19 | L87 | 26.57 | hydrophobic |
| L106 | 17.56 | F100 | 18.19 | hydrophobic |
| I108 | 18.03 | I101 | 20.26 | hydrophobic |
| F119 | 14.78 | G102 | 19.78 | hydrophobic |
| N61 | 15.83 | N40 | 18.82 | hydrogen bonding |
| R63 | 19.06 | R42 | 22 | hydrogen bonding |
| Q66 | 16.74 | Q45 | 21.69 | hydrogen bonding |
| **R79** | **17.79** | **R58** | **46.61** | **hydrogen bonding** |
| N94 | 15.85 | N73 | 25.7 | hydrogen bonding |
| N104 | 16.82 | N83 | 23.05 | hydrogen bonding |
| Q123 | 18.83 | Q104 | 20.14 | hydrogen bonding |

Amino acids are numbered as per the primary sequence of EcFbFP and iLOV. The listed values of B-factors reflect the highest B-factor associated with an amino acid. An alanine residue in the FMN-binding pocket that is involved in hydrophobic interactions with FMN (A62 in EcFbFP and A41 in iLOV) was excluded from consideration as the crystal structure used for EcFbFP (2PR5) belongs to the wild type YTVA protein wherein the A62 residue is replaced by the native C62. Therefore, a B-value for A62 in EcFbFP was not available. The arginine residue in iLOV with a particularly high B-factor (R58) is shown in bold.
