# Supplementary material for: Characterization of Flavin-Based Fluorescent Proteins: An Emerging Class of Fluorescent Reporters
Source: PLoS One. 2013 May 31;8(5):e64753. doi: 10.1371/journal.pone.0064753 (PMC3669411; doi:10.1371/journal.pone.0064753)
Supplement: Text S3 — Relative quantum yields of FbFPs as a function of pH, temperature, redox, and in anaerobic conditions. (DOCX) [file pone.0064753.s016.docx]

**Relative quantum yields of FbFPs as a function of pH, temperature, redox, and in anaerobic conditions**

We quantified the effects of pH, temperature, and reducing conditions on fluorescence from FbFPs in terms of the relative change in fluorescence emission at 495 nm, which corrsponds to the peak of the fluorescence emission spectrum under optimal conditions (25 °C, pH 6-7, 20 mM Tris hydrochloride, 1 M NaCl buffer). An alternative approach could involve calculating the relative change in quantum yield (ratio of integrated fluorescence emission spectrum to absorbance at excitation wavelength) as the temperature or pH is varied. In the **Tables S1-S3**, we enumerate relative quantum yields of the FbFPs up on incubation for 2.5 hours at various temperature and pH values as well as in 25 mM sodium dithionite (reductant). In addition, we incubated FbFPs in air-tight Balch tubes that had been deoxygenated by applying vacuum suction for 1 hour, thereby establishing an anaerobic environment. Relative quantum yields are calculated by dividing the integrated fluorescence emission spectra of the FbFPs at different temperature, pH, and under oxygen-depleted conditions respectively by the initial integrated emission spectra at room temperature (25 °C) or neutral pH (pH 6-7) or under oxygenated conditions (Balch tubes that had not been evacuated).

Importantly, we note the following caveat: relative quantum yields fail to precisely discriminate between contributions to the overall fluorescence emission spectrum from the FbFP-bound flavin and free flavin in solution. In particular, free flavin in solution has an emission spectrum with a peak at 525 nm and consequently contributes to the overall quantum yield, which is calculated by integrating the emission spectra between wavelength limits: 470 nm to 600 nm. Under optimal conditions of temperature and pH, concentration of free flavin is low enough to significantly contribute to the overall FbFP emission spectrum (**Supporting Text S1**). However, at high temperatures and extremes of pH, FbFPs tend to unfold, thereby liberating protein-bound flavin. As the concentration of free flavin in solution increases, the flavin spectrum contributes to the overall fluorescence quantum yield. In this way, relative quantum yields typically overestimate the net fluorescence from FbFPs. Therefore, we prefer to quantify the effects of environmental factors on FbFP fluorescence by comparing fluorescence values at the peak emission wavelength. In addition, we note that relative fluorescence emission at the peak wavelength has been widely employed for investigating the effects of environmental factors on protein fluorescence in case of several GFP-family proteins [1, 2, 3].

**Table S1. Relative quantum yields fof FbFPs as a function of temperature**

| **Protein** | **Rel. QY at 40 °C** | **Rel. QY at 50 °C** | **Rel. QY at 60 °C** | **Rel. QY at 70 °C** |
| --- | --- | --- | --- | --- |
| PpFbFP | 0.79 ± .03 | 0.58 | 0.57 | 0.53 |
| EcFbFP | 0.94 ± .03 | 0.85 ± .01 | 0.40 ± .09 | 0.23 |
| iLOV | 0.97 | 0.90 | 0.74 ± 0.02 | 0.32 |

FbFPs were incubated at the indicated temperatures for 2.5 hours and emission spectra were recorded between 470 and 600 nm following excitation at 450 nm. Relative (rel.) quantum yields were calculated by dividing the integrated emission spectra at the indicated temperatures by the emission spectrum at room temperature (25 °C).

**Table S2. Relative quantum yields fof FbFPs as a function of pH**

| **Protein** | **Rel. QY at pH 2** | **Rel. QY at pH 4** | **Rel. QY at pH 10** | **Rel. QY at pH 11** |
| --- | --- | --- | --- | --- |
| PpFbFP | 0.44 | 0.67 ± .02 | 0.60 | 0.25 |
| EcFbFP | 0.28 | 0.79 ± .01 | 0.92 ± .02 | 0.63 |
| iLOV | 0.29 | 0.74 ± .02 | 0.79 ± .02 | 0.58 ± .02 |

FbFPs were incubated at the indicated pH values for 2.5 hours and emission spectra were recorded between 480 and 600 nm following excitation at 450 nm. Relative (rel.) quantum yields were calculated by dividing the integrated emission spectra at the indicated pH values by the emission spectrum at pH 7 (EcFbFP and PpFbFP) or pH 6 (iLOV).

**Table S3. Relative quantum yields fof FbFPs in 25 mM sodium dithionite and under anaerobic conditions**

| **Protein** | **Rel. QY (25 mM dithionite)** | **Rel. QY (O_2_ depletion)** |
| --- | --- | --- |
| PpFbFP | 1.03 ± .02 | 1.00 ± .02 |
| EcFbFP | 1.03 | 0.98 ± .03 |
| iLOV | 0.99 ± .02 | 1.02 ± .02 |

FbFPs were incubated in reducing buffer (25 mM Tris hydrochloride, 1 M NaCl buffer supplemented with 25 mM sodium dithionite) for 2.5 hours or under oxygen-depletion conditions for 1.5 hours. Emission spectra were recorded between 480 and 600 nm following excitation at 450 nm. Relative (rel.) quantum yields were calculated by dividing the integrated emission spectra by the emission spectrum recorded in buffer lacking sodium dithionite or under oxygenated conditions.

**References**

1. Nagai T, Ibata K, Park ES, Kubota M, Mikoshiba K, et al. (2002) A variant of yellow fluorescent protein with fast and efficient maturation for cell-biological applications. Nat Biotech 20: 87-90.

2. Baird GS, Zacharias DA, Tsien RY. (2000) Biochemistry, mutagenesis, and oligomerization of DsRed, a red fluorescent protein from coral. PNAS 97(22): 11984-89.

3. Rizzo MA, Springer GH, Granada B, Piston DW. (2004) An improved cyan fluorescent protein useful for FRET. Nat Biotech 22(4): 445-449.

.
