# Supplementary material for: Characterization of Flavin-Based Fluorescent Proteins: An Emerging Class of Fluorescent Reporters
Source: PLoS One. 2013 May 31;8(5):e64753. doi: 10.1371/journal.pone.0064753 (PMC3669411; doi:10.1371/journal.pone.0064753)
Supplement: Text S4 — Nucleotide sequences of FbFP genes. (DOCX) [file pone.0064753.s017.docx]

**Nucleotide sequences of FbFP genes**

**>PpFbFP gene**

ATGATCAACGCAAAACTCCTGCAACTGATGGTCGAACATTCCAACGATGGCATCGTTGTCGCCGAGCAGGAAGGCAATGAGAGCATCCTTATCTACGTCAACCCGGCCTTCGAGCGCCTGACCGGCTACTGCGCCGACGATATTCTCTATCAGGACGCACGTTTTCTTCAGGGCGAGGATCACGACCAGCCGGGCATCGCAATTATCCGCGAGGCGATCCGCGAAGGCCGCCCCTGCTGCCAGGTGCTGCGCAACTACCGCAAAGACGGCAGCCTGTTCTGGAACGAGTTGTCCATCACACCGGTGCACAACGAGGCGGACCAGCTGACCTACTACATCGGCATCCAGCGCGATGTCACAGCGCAAGTATTCGCCGAGGAAAGGGTTCGCGAGCTGGAGGCTGAAGTGGCGGAACTGCGCCGGCAGCAGGGCCAGGCCAAGCACTGA

**>EcFbFP gene (codon optimized fro *E. coli*)**

ATGGCGTCGTTCCAGTCGTTCGGCATCCCGGGCCAGCTGGAAGTCATCAAGAAGGCGCTGGATCACGTGCGCGTCGGCGTGGTCATCACCGATCCCGCGCTGGAAGATAACCCGATCGTCTACGTGAACCAGGGCTTCGTGCAGATGACCGGCTACGAGACCGAGGAAATCCTGGGCAAGAACGCGCGCTTCCTCCAGGGGAAGCACACCGATCCGGCGGAAGTGGACAACATCCGCACCGCGCTGCAAAATAAAGAACCGGTCACCGTGCAGATCCAGAACTACAAGAAGGACGGCACGATGTTCTGGAACGAACTGAACATCGATCCGATGGAAATCGAGGATAAGACGTATTTCGTCGGCATCCAGAACGACATCACCAAGCAGAAGGAATATGAAAAGCTGCTCGAGTAA

**>iLOV gene (codon optimized fro *E. coli*)**

ATGATTGAAAAAAACTTTGTGATTACCGACCCGCGTCTGCCGGATAACCCGATCATTTTCGCGTCTGATGGCTTCCTGGAACTGACTGAGTATAGCCGTGAAGAAATCCTGGGCCGCAATGCTCGTTTTCTGCAGGGCCCGGAGACCGATCAAGCTACCGTGCAGAAGATTCGTGATGCAATTCGTGACCAGCGCGAAACGACTGTGCAGCTGATTAACTATACCAAGAGCGGTAAAAGATTCTGGAACCTGCTGCACCTGCAGCCGGTCCGTGATCAGAAAGGCGAGCTGCAGTATTTCATCGGTGTTCAGCTGGATGGTTCTGACCACGTATAA
